# Impact of IL-21 on CAR T-cell manufacturing: phenotypic, metabolic, and functional outcomes

**DOI:** 10.3389/fimmu.2026.1819376

**Published:** 2026-08-25

**Authors:** Maria Val-Casals, Ane Altuna, Lorena Pérez-Amill, Mercedes Armand-Ugón, Sergio Peña, Ana López-Pecino, Dolors Colomer, Jordi Esteve, Manel Juan, Nela Klein-González

**Affiliations:** 1 Fundació de Recerca Clínic Barcelona - Institut d’Investigacions Biomèdiques August Pi i Sunyer (FRCB-IDIBAPS), Barcelona, Spain; 2 Gyala Therapeutics S.L., Barcelona, Spain; 3 Faculty of Medicine and Health Sciences, Universitat de Barcelona, Barcelona, Spain; 4 Department of Immunology, Hospital Clínic de Barcelona, Barcelona, Spain; 5 Joint Platform of Advanced Therapies Hospital Sant Joan de Déu and Hospital Clínic de Barcelona, Esplugues de Llobregat, Spain; 6 Study Group for Immune Dysfunction Diseases in Children (GEMDIP), Institut de Recerca Sant Joan de Déu, Barcelona, Spain; 7 Hematopathology Section, Pathology Department, CDB, Hospital Clínic de Barcelona, Barcelona, Spain; 8 Centro de Investigación Biomédica en Red de Cáncer (CIBERONC), Madrid, Spain; 9 Department of Hematology, Hospital Clínic de Barcelona, Barcelona, Spain

**Keywords:** acute myeloid leukemia, CAR T cell, CAR T-cell manufacturing, CART84, CD84, cytokine modulation, cytokines, IL-21

## Abstract

**Introduction:**

Cytokines used during chimeric antigen receptor (CAR) T-cell manufacturing can influence product differentiation and functional fitness.

**Methods:**

We investigated whether adding IL-21 to an IL-7 + IL-15 expansion regimen modulates CD84-directed CAR T cells (CART84) targeting CD84^+^ acute myeloid leukemia (AML), using IL-7 + IL-15 alone as the primary comparator and IL-2 as a preclinical benchmark.

**Results:**

In healthy donor-derived CART84 cells, IL-21 supplementation was linked to an enrichment of less differentiated T-cell subsets, increased LAG-3^+^ CD8^+^ T-cell frequency, enhanced expansion following repeated antigen stimulation, and increased CAR expression under G-Rex-based expansion conditions more closely resembling GMP-compatible manufacturing. However, these effects were not accompanied by consistent improvements in cytotoxicity or sustained tumor control upon serial rechallenge *in vitro*. In AML patient-derived CART84 cells, IL-21 supplementation did not confer a clear advantage in expansion, T-cell memory phenotype, or cytotoxic activity. Furthermore, IL-21 did not enhance CART84 efficacy or persistence in a MOLM-13 AML xenograft model.

**Discussion:**

Overall, our results do not support routine incorporation of IL-21 into IL-7 + IL-15-based CART84 manufacturing protocols and highlight the need to evaluate cytokine supplementation strategies within the context of each CAR construct and disease setting.

## 1 Introduction

Chimeric antigen receptor (CAR) T-cell therapies have revolutionized the treatment landscape for hematologic malignancies, particularly B-cell malignancies (1). However, the efficacy and persistence of CAR T-cell therapies can be influenced by the phenotypic composition of the infused cells (2). T cells exist along a continuum of differentiation, ranging from naïve T cells (T_N_), which possess high proliferative capacity, to progressively differentiated subsets. As they differentiate, they give rise to stem cell memory T cells (T_SCM_), central memory T cells (T_CM_), effector memory T cells (T_EM_), and finally, terminally differentiated effector T cells (T_TE_), which are short-lived and prone to exhaustion. Early memory subsets — especially T_SCM_ — exhibit enhanced self-renewal, *in vivo* persistence, and long-term antitumor potential (3–5).

CAR T-cell products enriched in less differentiated subsets have frequently been associated with improved therapeutic outcomes (2). For instance, higher frequencies of early memory cells in infused CAR T-cell products correlated with improved clinical responses to CD19-directed CAR T-cell therapy (CART19) in chronic lymphocytic leukemia (CLL) (6), B-acute lymphoblastic leukemia (B-ALL) (7), and diffuse large B-cell lymphoma (DLBCL) (8, 9). Apheresis material and infusion products from responding patients have shown increased frequencies of T_SCM_-like cells across several CAR targets, including CD19 and BCMA, compared to non-responding patients (10–12). Moreover, memory-associated transcriptional programs are linked to CAR T-cell persistence (13, 14). Beyond efficacy, enrichment for less differentiated subsets has also been associated with reduced CAR T-cell–related toxicities in both preclinical (15) and clinical settings (9).

These observations have prompted efforts to refine CAR T-cell manufacturing to preserve therapeutically favorable phenotypes (16). Strategies include enriching early memory subsets prior to CAR transduction (10, 17–20), and modulating the cytokine environment during ex vivo expansion (21, 22). Interleukin-2 (IL-2), historically the most widely used cytokine, can drive T-cell proliferation and differentiation (23, 24). In contrast, IL-7 and IL-15 support robust expansion while more effectively maintaining memory-associated features and limiting excessive dysfunction (10, 24). Other γ-chain cytokines, such as IL-4, have also recently been explored in the context of CAR T-cell manufacturing to preserve T-cell fitness (25). Together, these data support cytokine modulation as a flexible lever in CAR T-cell manufacturing and motivate evaluation of additional γ-chain cytokines to further optimize CAR T-cell phenotype and function.

Interleukin-21 (IL-21) has more recently attracted interest as a cytokine capable of promoting stem-like properties and functional durability. During the priming and activation of antigen-specific CD8^+^ T cells, IL-21 favors the generation and maintenance of memory-stem–like populations with enhanced proliferative capacity and antitumor function (26–29). These effects have been linked to metabolic remodeling toward mitochondrial oxidative phosphorylation (OXPHOS), consistent with improved mitochondrial fitness and long-term cellular function (26, 30–32). Beyond adoptive cell transfer, IL-21–based interventions are reported to enhance antitumor activity in preclinical immune checkpoint blockade models (33). In the CAR T-cell context, IL-21 has been shown to limit terminal differentiation, support the expansion of T_SCM_ cells and increase proliferative capacity, with evidence from preclinical studies indicating improved tumor control (17, 32, 34–42). Its integration into clinical CAR T-cell manufacturing remains at an early stage and is still under active evaluation (43, 44).

In the context of CAR T-cell therapy for acute myeloid leukemia (AML), it is important to consider that T cells from AML patients are frequently dysfunctional and exhibit features of exhaustion, partly driven by the immunosuppressive tumor microenvironment (45, 46). This underscores the need to improve T-cell fitness during manufacturing when starting from patient-derived (autologous) material. Alongside these constraints, the identification of suitable target antigens remains a challenge in AML. Unlike B-cell malignancies, for which several CAR T-cell therapies are approved, no CAR T-cell product has yet been approved for AML, largely due to its biological heterogeneity and the lack of an optimal leukemia-specific antigen (47). CD84, also known as Signaling Lymphocytic Activation Molecule Family (SLAM) member 5, is a homophilic surface glycoprotein of the SLAM family. It is broadly expressed on hematopoietic cells—particularly monocytes, memory B and T cells, and platelets—where it contributes to monocyte activation, T-B cell interactions, and B-cell tolerance in germinal centers (48). CD84 has recently been identified as a therapeutic target in AML (49, 50), and CAR T cells directed against CD84 (CART84) provide a new treatment approach (51, 52). CART84 is currently under clinical evaluation at our institution in Spain (EudraCT 2024-519966-31–00 and NCT07471789) and China (NCT06786299) for the treatment of AML.

In this study, we evaluate the effect of IL-21—in combination with other γ-chain cytokines— on CART84 differentiation and function. We hypothesize that IL-21 promotes a less differentiated state with enhanced persistence and antitumor activity *in vivo*. These findings may guide cytokine selection during the manufacture of CAR T-cell therapies targeting AML.

## 2 Materials and methods

### 2.1 Cell lines

HL-60/S4 (Cat#CRL-3306, RRID: CVCL_II77) and U-937 (Cat#CRL-1593.2, RRID: CVCL_0007) AML cell lines were obtained from American Type Culture Collection (ATCC); MOLM-13 AML cell line (Cat#ACC 554, RRID: CVCL_2119) was obtained from Deutsche Sammlung von Mikroorganismen und Zellkulturen (DSMZ); K-562 (chronic myelogenous leukemia in blast crisis, RRID: CVCL_0004) and HEK293T (RRID: CVCL_0063) cells were kindly provided by Dr. Manel Juan (Hospital Clínic de Barcelona, HCB); and MOLT-4 (RRID: CVCL_0013) by Dr. Pablo Menéndez (Josep Carreras Leukemia Research Institute). Suspension leukemia lines were cultured in RPMI-1640 (Cat#21875091) supplemented with 10% heat-inactivated FBS (Cat#F9665), GlutaMAX (Cat#35050061), and penicillin/streptomycin (Cat#15140-122), and HEK293T in DMEM (Cat#10313021) with the same supplements (all from Gibco). GFP/firefly luciferase (GFP-ffLuc) reporter lines were generated by lentiviral transduction (using the pLV430GoFL-T2A-GFP plasmid, kindly provided by the University of Texas, M.D. Anderson Cancer Center). Cell line identity was confirmed by short tandem repeat profiling (qGenomics) and routine mycoplasma testing was performed.

### 2.2 Generation of CD84-directed CAR T cells

The CAR84 construct and CAR manufacturing workflow have been described previously (51). Briefly, the anti-CD84 scFv (153-4D9 V_H_/V_L_ linked by a (G4S)_4_ linker) was cloned into a second-generation lentiviral CAR containing a CD8α hinge, 4-1BB costimulatory domain, and CD3ζ signaling domain under the EF1α promoter (pCCL_EF1α backbone). Lentiviral particles were produced by transient transfection of HEK293T cells with the CAR transfer plasmid and standard packaging/envelope plasmids (pRSV-Rev (Addgene, Cat#12253; RRID: Addgene_12253), pMDLg/pRRE (Addgene, Cat#12251; RRID: Addgene_12251), pMD2.G (Addgene, Cat#12259; RRID: Addgene_12259)) in the presence of polyethylenimine (Polysciences, Cat#23966-2). Viral supernatants were collected at 48 and 72 h, concentrated using Lenti-X concentrator (Takara, Cat# 631232), titrated by limiting dilution on HEK293T cells using streptavidin-based CAR detection, and stored at −80 °C.

Approval for the use of human samples was obtained from the Clinical Research Ethics Committee of Hospital Clínic de Barcelona (protocols HCB/2021/0977 and HCB/2024/0309). T cells from healthy donor buffy coats (Banc de Sang i Teixits) were enriched by negative selection (RosetteSep™ Human T Cell Enrichment Cocktail, STEMCELL Technologies, Cat#15061); T cells from AML patient samples (**Supplementary Table 1**) were isolated by positive selection using CD3 MicroBeads (Miltenyi Biotec Cat#130-097-043). T cells were activated with anti-CD3/CD28 beads (Gibco, Cat#11132D) in X-VIVO™ 15 medium (Lonza, Cat#H3BE02-060F), supplemented with 5% heat-inactivated human serum (Merck Millipore, Cat#H4522), 1% GlutaMAX and 1% penicillin/streptomycin.

Cells were expanded under one of three cytokine conditions: (1) a standard preclinical condition utilizing only IL-2 (5 ng/mL, Miltenyi Biotec Cat#130-097-748, equivalent to 50 IU/mL as previously described) (IL2) (51, 53); (2) a condition designed to simulate standard good manufacturing practices (GMP) settings used at our institution, employing IL-7 (12.5 ng/mL, Miltenyi Biotec Cat#130-095-363) and IL-15 (12.5 ng/mL, Miltenyi Biotec Cat#130-095-765), equivalent to 155 IU/mL IL-7 and 290 IU/mL IL-15 used in closed-system bioreactors (CliniMACS Prodigy, Milteny Biotech) (IL7 + 15) (53–57); and (3) the GMP-aligned condition further supplemented with IL-21 (30 ng/mL, Miltenyi Biotec Cat#130-095-767), a concentration selected based on prior literature demonstrating its effects on CAR T-cell differentiation (IL7 + 15 + 21) (36, 37, 44).

Cells were transduced 24 h after activation, or 48 h after activation for cryopreserved samples, at a Multiplicity of Infection (MOI) of 5 in the presence of polybrene (Merck Millipore, Cat#TR-1003-G) by spinoculation (2,000 rpm, 1 h). Medium was replaced after 6 h, and activation beads were removed 5 days after transduction. CART84 products were harvested on day 7 post-transduction, corresponding to day 8 of culture for fresh samples and day 9 for cryopreserved samples, and maintained in their respective cytokine conditions throughout expansion.

To evaluate the effect of IL-21 supplementation under manufacturing conditions more closely resembling GMP-compatible bioreactor systems, CART84 cells were additionally assessed in gas-permeable rapid expansion (G-Rex) culture vessels. T cells isolated from healthy donor buffy coats were seeded in TexMACS™ medium (Miltenyi Biotec, Cat#130-097-196) supplemented with 5% heat-inactivated human serum and stimulated with T-cell TransAct (Miltenyi Biotec, Cat#130-111-160) for 24 h in G-Rex 24-well plates (Wilson Wolf, Cat#80192M). Cells were cultured in either IL-7 (12.5 ng/mL) plus IL-15 (12.5 ng/mL) (IL7 + 15) or IL-7 + 15 supplemented with IL-21 (30 ng/mL) (IL7 + 15 + 21), with cytokines added at the time of stimulation. Twenty-four hours later, cells were transduced at a MOI of 5 without spinoculation. An additional 24 h later, 7 mL of fresh medium was added per well, followed by a partial medium exchange on day 5 post-transduction.

### 2.3 Cytotoxicity assays

Target cells were incubated with CART84 at the indicated effector-to-target (E:T) ratios for 24 hours in 96-well round-bottom plates. For GFP-ffLuc-expressing targets, live GFP^+^ cells were quantified by flow cytometry and normalized to target-only controls to calculate residual viability. HL-60/S4 targets were labeled with CFSE (Invitrogen, Cat#C34554) prior to co-culture and quantified as live CFSE^+^ cells. Cytotoxicity was calculated using the following formula: % of live cells = (number of target cells with effector cells at 24 h/number of target cells alone at 24 h) x 100.

### 2.4 Cytokine production

Cell-free supernatants were harvested 24 h after co-culture with MOLM-13 at an E:T ratio of 1:2 to assess CART84 cytokine secretion. TNFα, IL-2, IFNγ and granzyme B cytokines were quantified by enzyme-linked immunosorbent assay (ELISA, R&D Systems, Cat#DY210-05, Cat#DY202-05, Cat# DY285B-05 and Cat# DY2906-05) according to manufacturer’s instructions.

### 2.5 Metabolic profiling using Seahorse XF analyzer

Thawed CART84 were rested overnight in cytokine-supplemented medium and analyzed using the Seahorse T Cell Metabolic Profiling Kit (Agilent Cat#103772-100). Cells (1×10^5^/well) were plated on PDL-coated Seahorse plates (Cat# 103727–100 and Cat# 103799-100) in Seahorse XF assay medium (Cat#103681-100) (all from Agilent). Oxygen consumption rate (OCR) was measured on Seahorse XF HS Mini or XF Pro analyzers (Agilent) following sequential injection of mitochondrial inhibitors (including oligomycin, BAM15 and rotenone) according to the manufacturer’s instructions for T-cell persistence assay.

### 2.6 Repeated antigen stimulation assay

MOLM-13-GFP-ffLuc cells were irradiated (30 Gy) and co-cultured with thawed CART84 at a 1:1 E:T ratio (1×10^6^ cells each; 0.5×10^6^ cells/mL). Every 2–3 days, cultures were sampled for CD3^+^ quantification and replenished with irradiated MOLM-13-GFP-ffLuc to maintain E:T ratio and volume; media exchange and/or dilution was performed as needed to keep cell density ≤1×10^6^ cells/mL. CD3^+^ cell counts were quantified by flow cytometry, and a portion of the cells was also collected for immunophenotyping as described below.

### 2.7 Long-term *in vitro* tumor rechallenge assay

CART84 cells were co-cultured with non-irradiated MOLM-13-GFP-ffLuc target cells at E:T ratio of 1:4 (0.25×10^6^ CART84 cells and 1×10^6^ MOLM-13-GFP-ffLuc cells). Upon tumor elimination, 1×10^6^ non-irradiated MOLM-13-GFP-ffLuc target cells were added without removing effector cells. Rechallenges continued until loss of antitumor activity. At the indicated time points, fixed-volume samples were collected for flow cytometric quantification of GFP^+^ and CD3^+^ cells, and cell numbers were normalized to the total culture volume.

### 2.8 Flow cytometry and immunophenotyping

CART84 immunophenotyping was performed using surface antibodies against CD3, CD4, CD8, CD45RA/RO, CD62L, CD69, CD84, CD95, PD-1, TIM-3, LAG-3, and CTLA-4 (BioLegend and BD Biosciences). Fluorescence minus one (FMO) controls were used to define positivity thresholds and applied consistently across samples from the same donor. T-cell memory subsets were defined as shown in **Supplementary Figure 1**. Activation, exhaustion, and co-expression analyses were performed within CD4^+^ and CD8^+^ T-cell populations as illustrated in **Supplementary Figures 2**, **3**. CAR expression was detected using biotinylated anti-mouse F(ab’)2 (Jackson ImmunoResearch) followed by streptavidin conjugates (eBioscience and BD Biosciences). Samples were acquired on LSRFortessa™ (BD Biosciences) or CytoFLEX (Beckman Coulter) flow cytometers using consistent instrument settings within each donor, and analyzed using FlowJo v10 (BD Biosciences, RRID: SCR_008520). For SPICE analysis (SPICE v6.1; RRID: SCR_016603), FlowJo gating was performed as described in **Supplementary Figure 2**, and combinatorial PD-1, LAG-3, and TIM-3 expression gates were exported to SPICE for visualization and analysis (74). Antibody catalog numbers and RRIDs are provided in **Supplementary Table 2**.

### 2.9 *In vivo* xenograft mouse model

All animal procedures were approved by the Animal Experimentation Ethics Committee of the Generalitat de Catalunya, under protocol number 11577. Animals were housed under specific pathogen-free conditions at the University of Barcelona Animal Facility. On day 0, 1×10^5^ MOLM-13-GFP-ffLuc cells suspended in 100 μL of saline solution (Braun Cat#607189.2) were administered intravenously (i.v.) via the tail vein to immunodeficient NSG (NOD-Cg-Prkdcscid-Il2rgtm1Wjl/SzJ, RRID: IMSR_JAX:005557) female or male (**Supplementary Figure 9**) mice (Charles River). On day 2, mice were allocated to experimental groups based on tumor burden to ensure similar baseline tumor loads. Subsequently, 5x10^6^ fresh or cryopreserved (**Supplementary Figure 9**) CAR^+^ CART84 cells, suspended in 100 μL of saline solution, were administered i.v.

To monitor leukemia burden, 100 μL of luciferin (20 mg/mL, Revvity, Cat#122799) was administered intraperitoneally to the mice. The mice were then anesthetized with 2% inhaled isoflurane (Zoetis Spain) and imaged for bioluminescence using a Xenogen IVIS 50 system with Living Image v4.74 software (PerkinElmer). When the humane endpoint criteria or the experimental endpoint were reached, mice were anesthetized with isoflurane (2% inhalation) and euthanized by cervical dislocation performed by trained personnel. Bone marrow samples were collected at the time of euthanasia, flushed, and analyzed by flow cytometry following RBC lysis (ACK buffer; Gibco, Cat#A1049201) using antibodies against mouse CD45 and human CD45/CD3 (BD Biosciences; antibodies listed in **Supplementary Table 2**).

### 2.10 Statistical analysis

Statistical analyses were performed using GraphPad Prism 10.4.0 (RRID: SCR_002798). Sample sizes and statistical analyses tests are detailed in the corresponding figure legends. Two-group comparisons were performed using t-tests, whereas multi-group comparisons were analyzed using ANOVA or mixed-effects models. Matching strategies and multiple-comparison corrections were applied as indicated in the corresponding figure legends. Statistical significance was defined as *p* < 0.05.

### 2.11 Ethics statement

#### 2.11.1 Human samples

The use of human samples was approved by the Clinical Research Ethics Committee of Hospital Clínic de Barcelona (protocols HCB/2021/0977 and HCB/2024/0309). Buffy coats were obtained from healthy donors through the local blood bank (Banc de Sang i Teixits). Primary acute myeloid leukemia (AML) samples were obtained from patients diagnosed with AML and treated at Hospital Clínic de Barcelona. Written informed consent for research use of samples was obtained from all healthy donors and patients, in accordance with the Declaration of Helsinki and applicable institutional and national regulations.

#### 2.11.2 Animal studies

All animal procedures were approved by the Animal Experimentation Ethics Committee of the Generalitat de Catalunya (protocol no. 11577). Animal experiments were conducted in accordance with institutional guidelines and in compliance with applicable national and European legislation for the care and use of laboratory animals, including Directive 2010/63/EU. All efforts were made to minimize animal suffering and to reduce the number of animals used.

## 3 Results

### 3.1 IL-21 influences CART84 T-cell differentiation during *ex vivo* expansion

To determine whether cytokine composition modulates T-cell differentiation during CAR T-cell expansion, CART84 cells were grown under three distinct conditions: (1) a standard preclinical condition utilizing only IL-2 (5 ng/mL, equivalent to 50 IU/mL as previously described) (IL2) (51, 53); (2) a condition designed to simulate standard good manufacturing practices (GMP) settings used at our institution, employing IL-7 (12.5 ng/mL) and IL-15 (12.5 ng/mL), equivalent to 155 IU/mL IL-7 and 290 IU/mL IL-15 used in closed-system bioreactors (IL7 + 15) (53–57); and (3) the GMP-aligned condition further supplemented with IL-21 (30 ng/mL, Cat#130-095-767), a concentration selected based on prior literature demonstrating its effects on CAR T-cell differentiation (IL7 + 15 + 21) (36, 37, 44).

**Figure 1:**
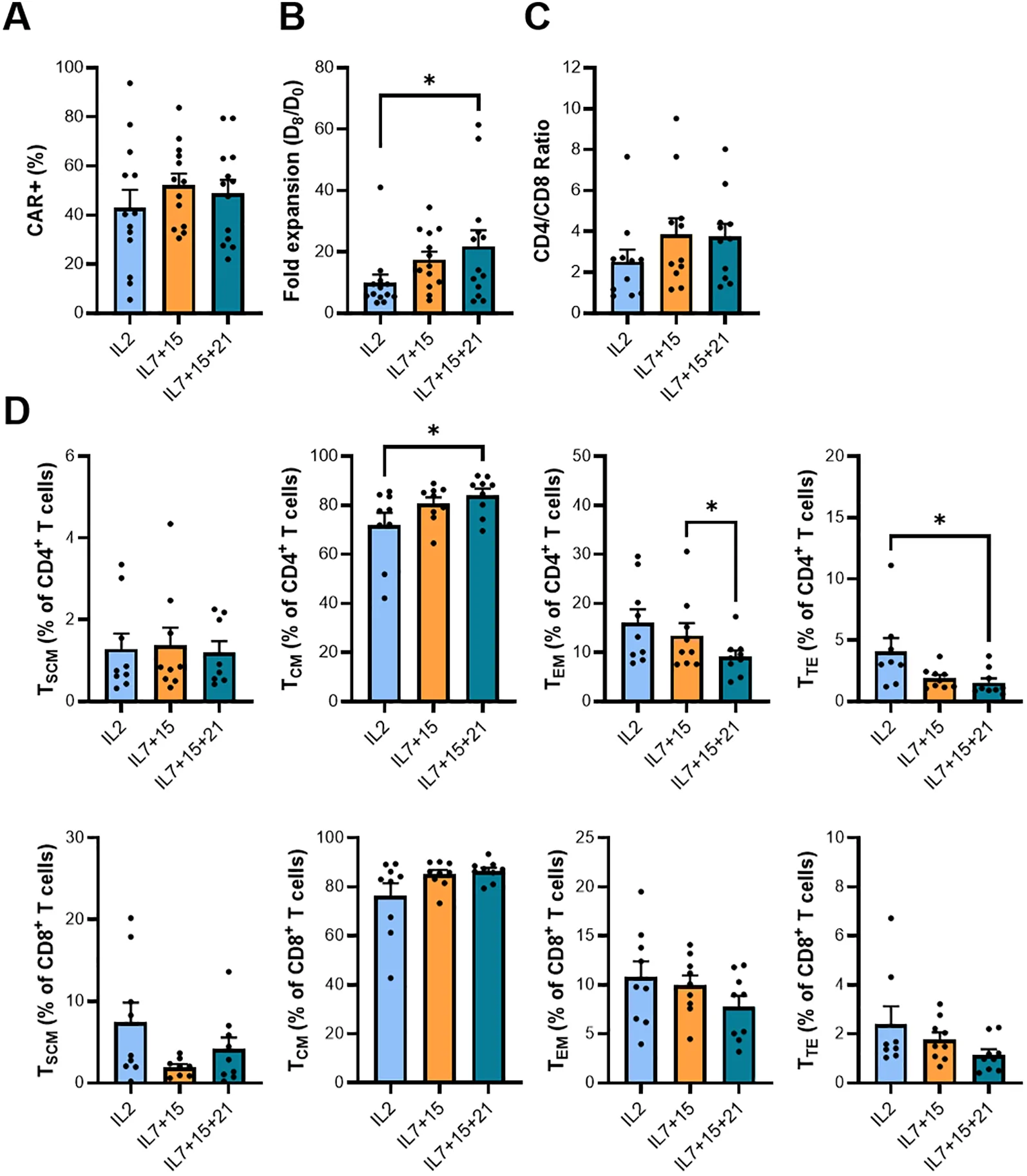
Characterization of CART84 expanded under different cytokine conditions. **(A)**Percentage of CAR^+^ cells. Data represent 13 independent donors (n = 13), mean ± S.E.M. **(B)** Fold expansion. Data represent 13 independent donors (n = 13), mean ± S.E.M. **(C)** CD4/CD8 ratio. Data represent 11 independent donors (n = 11), mean ± S.E.M. Statistical analysis was performed using one-way ANOVA with donor-matched cytokine conditions (IL-2, IL-7 + 15 and IL-7 + 15 + 21) followed by Tukey’s post-test for multiple comparisons. *: *p* < 0.05. Comparisons not reaching significance are not shown. **(D)** Characterization of T-cell memory subsets. Naïve T cells (T_N_), stem cell memory T cells (T_SCM_), central memory T cells (T_CM_), effector memory T cells (T_EM_), and terminal effector T cells (T_TE_) were identified within CD4^+^ (top row) and CD8^+^ (bottom row) T cells using the gating strategy shown in **Supplementary Figure 1**. T_N_ cells are not shown due to their negligible frequency at this time point. Outliers were identified using the ROUT method (Q = 1%) and excluded from subsequent analyses. Statistical analysis was performed using one-way ANOVA, or mixed-effects models when values were missing, with donor-matched cytokine conditions (IL-2, IL-7 + 15 and IL-7 + 15 + 21), followed by Tukey’s post-test for multiple comparisons. Data represent 9 independent donors (n = 9), mean ± S.E.M. *: *p* < 0.05. Comparisons not reaching significance are not shown.

CAR expression was comparable across all cytokine conditions (**Figure 1A**). On average, IL-7 + 15 + 21 modestly increased CART84 fold expansion compared with IL-2, an effect observed in the majority of donors (10/13). IL-21 supplementation did not further enhance fold expansion compared with IL-7 + 15 alone (**Figure 1B**). CD4/CD8 ratios remained similar across all conditions (**Figure 1C**).

**Figure 2:**
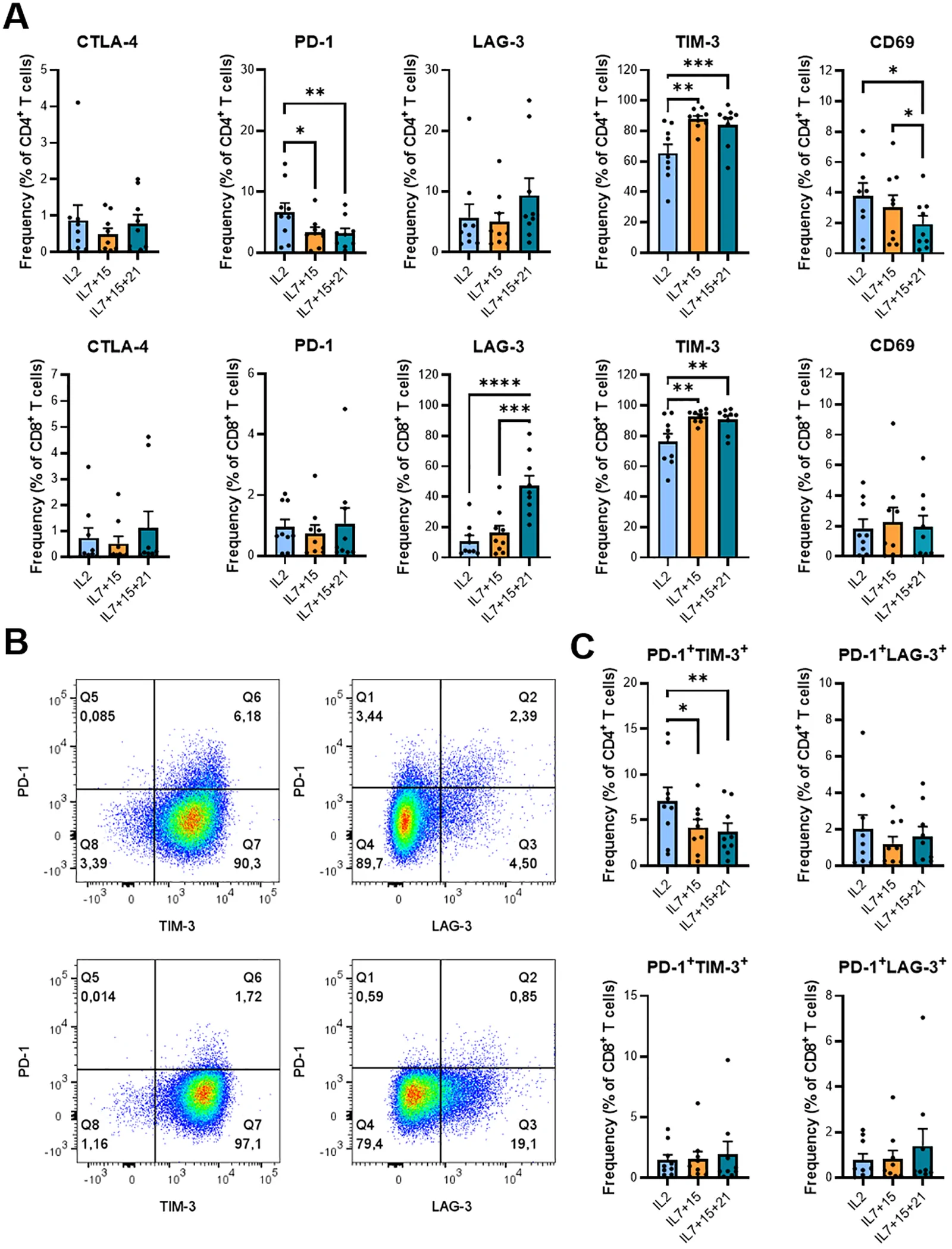
Expression of exhaustion and activation markers in CART84 expanded under different cytokineconditions. **(A)** Percentage of cells positive for the indicated exhaustion andactivation markers within CD4^+^ (top row) and CD8^+^ (bottom row) T-cellcompartments, following the gating strategy shown in **Supplementary Figure 2**. **(B)** Representative gating plots for PD-1^+^TIM-3^+^ and PD-1^+^LAG-3^+^ cells within CD4^+^ (top row) and CD8^+^ (bottom row) T cells. The full gating strategy is shown in **Supplementary Figure 3**. (C) Percentage of PD-1^+^TIM-3^+^ or PD-1^+^LAG-3^+^ double-positive cells within CD4^+^ (top row) and CD8^+^ (bottom row) T cells. Gates for marker positivity were defined using the corresponding fluorescence minus one (FMO) controls. Statistical analysis was performed using one-way ANOVA with donor-matched cytokine conditions (IL-2, IL-7 + 15 and IL-7 + 15 + 21) followed by Tukey’s post-test for multiple comparisons. Data represent 9 independent donors (n = 9; same donors across all panels), mean ± S.E.M. *: *p* < 0.05, **: *p* < 0.01, ***: *p* < 0.001, ****: *p* < 0.0001. Comparisons not reaching significance are not shown.

Motivated by prior reports indicating that IL-21 facilitates the persistence of stem-cell memory CAR T-cells during *in vitro* expansion (32, 35, 36), the T-cell memory phenotype of CD4^+^ and CD8^+^ CART84 cells was characterized throughout the expansion using flow cytometry (**Supplementary Figure 1**). Kinetics were comparable across conditions: upon activation (day 0), T_N_ cells rapidly differentiated into T_SCM_, peaking between days 2 and 3 before declining. T_CM_ cells progressively accumulated and stabilized around day 5, whereas T_EM_ cells decreased until day 5 with a slight post-debeading increase. T_TE_ cells remained low throughout expansion (**Supplementary Figure 4**).

At the end of the expansion (day 8), IL-21 partially reshaped the memory composition of the CD4^+^ compartment, as it was associated with increased frequency of T_CM_ cells compared to the preclinical IL-2 condition, with a consistent effect observed across donors (n = 9). This was accompanied by a reduction in T_EM_ compared to IL-7 + 15 alone, and in T_TE_ cells compared to IL-2, observed in n = 9 and n = 8 donors, respectively. IL-21 addition did not significantly increase the frequency of T_SCM_ in CART84 (**Figure 1D**). No significant differences were observed in CD8^+^ T-cell populations (n = 9).

### 3.2 Effect of cytokine conditions on exhaustion and activation marker expression in CART84

Expression of key inhibitory receptors CTLA-4, PD-1, LAG-3, TIM-3, and the activation marker CD69 was analyzed in CART84 (**Supplementary Figure 2**), given prior links between exhaustion marker expression and reduced clinical efficacy (6, 9, 58). Overall, no consistent pattern was observed across cytokine conditions, and phenotypic differences were generally limited (**Figure 2A**). In the CD4^+^ compartment, CART84 cells expanded in IL-7 + 15, with or without IL-21 supplementation, displayed lower frequencies of PD-1^+^ cells and higher frequencies of TIM-3^+^ cells compared with IL-2-expanded CART84. IL-21 supplementation also reduced the frequency of CD69^+^ CD4^+^ T cells. In the CD8^+^ compartment, IL-7 + 15-expanded CART84 cells (regardless of IL-21) similarly showed higher frequencies of TIM-3^+^ expressing cells compared to IL-2, whereas the main effect associated with IL-21 supplementation was an increase in the frequency of LAG-3^+^ cells. These differences were consistently observed across donors (n = 9), although their magnitude varied between donors.

PD-1^+^TIM-3^+^ and PD-1^+^LAG-3^+^ double-positive populations, previously associated with worse clinical responses in CART19-treated CLL patients (6), were also analyzed (**Supplementary Figure 3**). Overall, cytokine conditions did not significantly affect these subsets, except for a reduction in PD-1^+^TIM-3^+^ CD4^+^ T cells under IL-7 + 15, independently of IL-21, consistently observed across donors (n = 9) (**Figures 2B, C**).

To further characterize exhaustion marker single and co-expression patterns, PD-1, TIM-3, and LAG-3 expression was analyzed using SPICE (**Supplementary Figure 5**). In CD4^+^ CART84 cells, IL-21 supplementation did not significantly alter the distribution of PD-1, TIM-3, and LAG-3 expression patterns compared with IL-7 + 15 alone. Both IL-7 + 15 and IL-7 + 15 + 21-expanded CD4^+^ T cells showed different patterns compared to IL-2, likely reflecting the higher frequency of single TIM-3-expressing cells in the former conditions (**Supplementary Figure 5A**). In contrast, CD8^+^ CART84 cells displayed distinct co-expression profiles across all cytokine conditions. The distinct profile observed in IL-7 + 15 + 21-expanded CD8^+^ CART84 cells appeared to be driven by a greater representation of TIM-3^+^LAG-3^+^ co-expressing populations compared with IL-7 + 15-expanded CART84 cells. Differences between IL-7 + 15-based conditions and IL-2-expanded CART84 cells were primarily associated with a higher representation of TIM-3^+^ populations in the former conditions (**Supplementary Figure 5B**).

### 3.3 Cytotoxic activity and antigen-induced cytokine secretion in CART84 under different cytokine conditions

**Figure 3:**
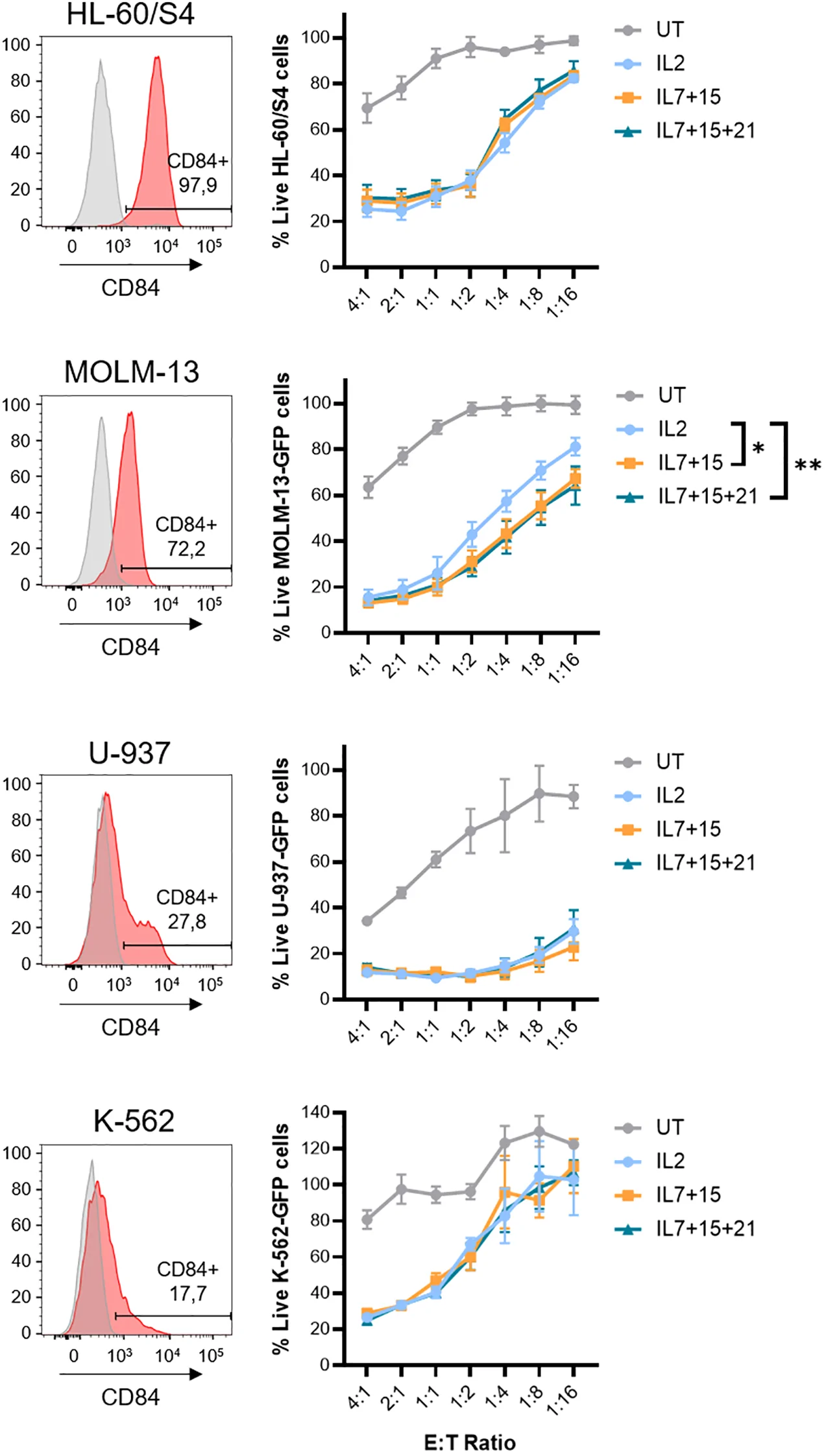
*In vitro* cytotoxicity of CART84 expanded under different cytokine conditions. Untransduced (UT) T cells and CART84 generated under the indicated cytokine conditions were co-cultured with the indicated target cell lines for 24 h at the indicated effector-to-target (E:T) ratios. On the left, flow cytometry histograms of CD84 expression (red) relative to isotype control (grey) are shown for each cell line. Data represent 3–13 independent donors, depending on the target cell line: HL-60/S4 (n = 3–6), MOLM-13 (n = 3–13), K-562 (n = 3–6), and U-937 (n = 2–8). Cytotoxicity was analyzed using two-way ANOVA followed by Tukey’s post-test for multiple comparisons. Only CART84 cells expanded under different cytokine conditions were included in the statistical analysis (UT excluded). Enhanced cytotoxicity was observed against MOLM-13 cells in CART84 expanded with IL-7 + 15 compared with IL-2-expanded CART84, particularly at lower E:T ratios, whereas IL-21 supplementation did not confer a consistent additional benefit. Mean ± S.E.M. *: *p* < 0.05; **: *p* < 0.01. Comparisons not reaching significance are not shown.

Cytotoxic activity of CART84 cells expanded under different cytokine conditions was evaluated against AML cell lines expressing different levels of CD84: HL-60/S4 and MOLM-13 (high CD84), and U-937 and K-562 (moderate/low CD84). CART84 mediated cytotoxicity against all target cell lines, and killing did not strictly correlate with CD84 expression levels, as U-937, despite expressing intermediate CD84 levels, showed levels of CART84-mediated cytotoxicity comparable to those observed in the high-CD84 cell lines. The addition of IL-21 did not further enhance cytotoxic activity, as IL-7 + 15 and IL-7 + 15 + 21-expanded CART84 displayed comparable cytotoxicity across all target cell lines. Compared with IL-2-expanded CART84, both IL-7 + 15-based expansion conditions showed greater killing of MOLM-13, particularly at low effector-to-target (E:T) ratios (1:2–1:16) (**Figure 3**).

Cytotoxicity was also evaluated against the T-acute lymphoblastic leukemia (T-ALL) cell line MOLT-4, which expresses high levels of CD84. Cytotoxic activity was comparable across all expansion conditions (**Supplementary Figure 6**).

To determine whether enhanced MOLM-13 killing by IL-7 + 15 ± IL21–expanded CART84 was associated with increased cytokine secretion, we quantified TNFα, IL-2, IFNγ and granzyme B in 24-hour co-culture supernatants from 6 donors by ELISA (**Supplementary Figure 7**). IL-7 + 15–expanded CART84 displayed increased TNFα secretion relative to IL-2–expanded CART84, and increased IL-2 secretion compared with both IL2– and IL-7 + 15 + 21–expanded CART84; a pattern consistently observed across all donors (n = 6). IFNγ and granzyme B secretion were not significantly affected by the cytokine regimen. Although both IL-7 + 15 and IL-7 + 15 + 21 conditions enhanced MOLM-13 killing, IL-21 did not further increase cytokine secretion (**Supplementary Figure 7**).

### 3.4 Effect of IL-21 supplementation on the metabolic profile of CART84

**Figure 4:**
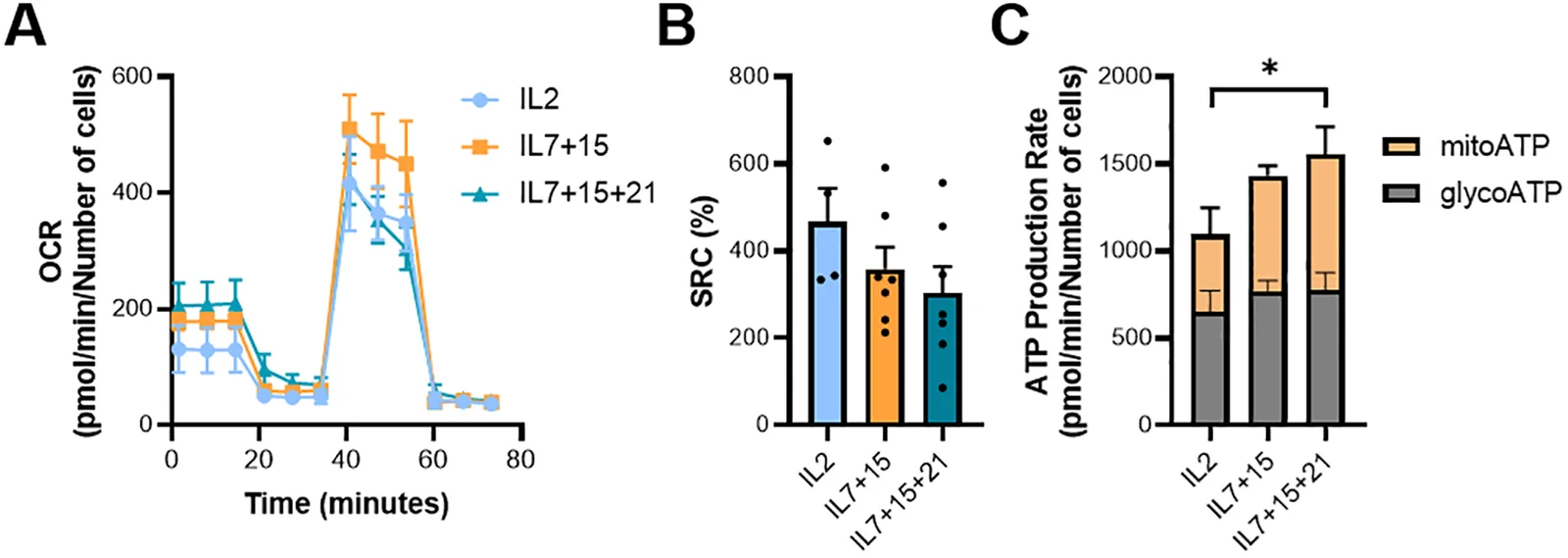
Metabolic profiling of CART84 expanded under different cytokine conditions. **(A)** Oxygen consumption rate (OCR) of CART84 expanded with different cytokines in a T-cell persistence Seahorse assay. Statistical analysis was performed using mixed-effects models with donor-matched cytokine conditions (IL-2, IL-7 + 15, and IL-7 + 15 + 21) and time, followed by Tukey’s post-test for multiple comparisons. **(B)** Spare respiratory capacity (SRC) of CART84 expanded with different cytokines. Statistical analysis was performed using mixed-effects models with donor-matched cytokine conditions (IL-2, IL-7 + 15 and IL-7 + 15 + 21) followed by Tukey’s post-test for multiple comparisons. **(C)** Basal ATP production rate from OXPHOS (mitoATP) or glycolysis (glycoATP) of CART84 expanded with different cytokines. Statistical analysis was performed using mixed-effects models with donor-matched cytokine conditions (IL-2, IL-7 + 15, and IL-7 + 15 + 21) and ATP production pathways (mitoATP and glycoATP), followed by Tukey’s post-test for multiple comparisons. Data represent 4–7 independent donors (n = 4 independent donors for IL-2, n = 7 independent donors for IL7 + 15 and IL7 + 15 + 21, same donors across all panels), mean ± S.E.M. *: *p* < 0.05. Comparisons not reaching significance are not shown.

T-cell differentiation states are associated with distinct metabolic profiles. Less differentiated T-cell subsets (T_SCM_ and T_CM_) rely primarily on mitochondrial oxidative metabolism, exhibiting higher basal OXPHOS activity, mitochondrial ATP (mitoATP) production, and spare respiratory capacity (SRC). In contrast, more differentiated effector T cells (T_EM_ and T_TE_) rely more strongly on glycolytic metabolism (59, 60). CART84 cells expanded under different cytokine conditions were analyzed for metabolic activity using a Seahorse XF Analyzer (Agilent Technologies). Oxygen consumption rate (OCR) kinetics (**Figure 4A**), and SRC were comparable across all conditions (**Figure 4B**). CART84 cells expanded in the presence of IL-21 showed increased basal mitoATP production compared with IL-2-expanded CART84 cells. However, no significant differences in basal mitoATP production were observed between IL-7 + 15- and IL-7 + 15 + 21-expanded CART84 cells, indicating that IL-21 supplementation did not further alter the metabolic profile of CART84 cells expanded under IL-7 + 15-based conditions (**Figure 4C**).

### 3.5 Impact of IL-21 on CART84 expansion upon repeated antigen stimulation

**Figure 5:**
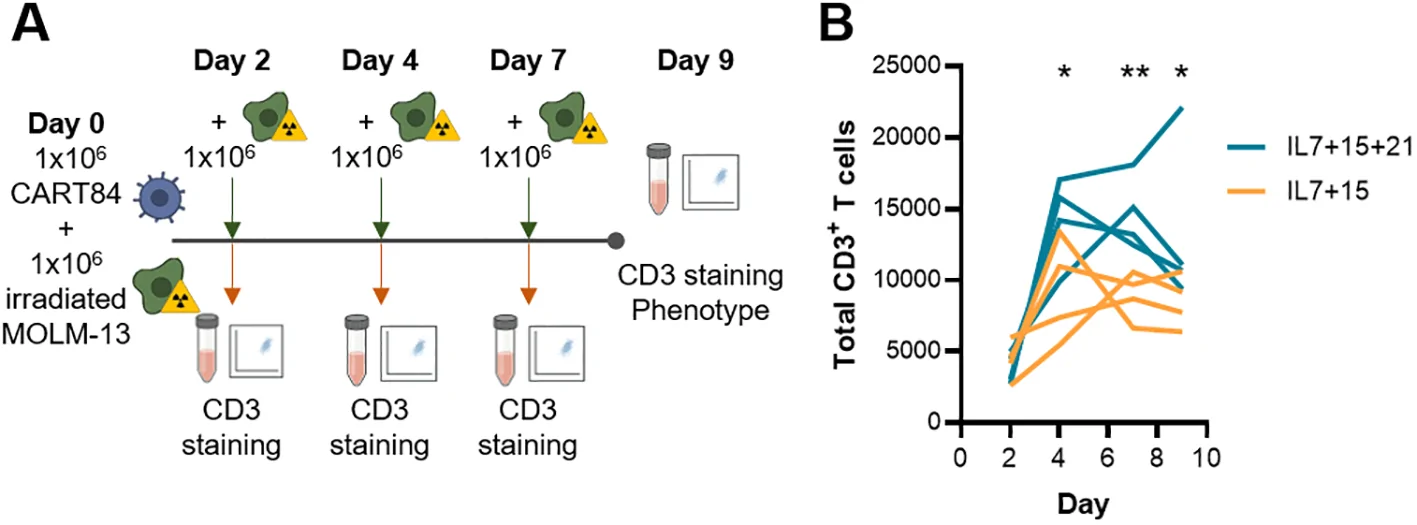
Proliferative capacity of CART84 expanded under different cytokine conditions upon repeated antigen exposure. **(A)** Experimental design. **(B)** CD3^+^ cell counts at the indicated time points. After each time point, CART84 were rechallenged with irradiated MOLM-13 cells at an initial effector-to-target ratio of 1:1. Statistical analysis was performed using two-way ANOVA with donor-matched cytokine conditions (IL-7 + 15 and IL-7 + 15 + 21) and time points, followed by Sídák’s post-test for multiple comparisons. Data represent 4 independent donors (n = 4), mean ± S.E.M. *: *p* < 0.05, **: *p* < 0.01. Comparisons not reaching significance are not shown.

Subsequent functional assays focused on IL-7 + 15 with or without IL-21. To determine whether differences in cytokine conditions translated into improved proliferation, CART84 cells expanded under IL-7 + 15 ± IL-21 were subjected to repeated antigen stimulation. Cells were co-cultured with irradiated MOLM-13 cells at an E:T ratio of 1:1 every 2–3 days to monitor CAR T-cell proliferation over time (**Figure 5A**). CART84 cells grown with IL-21 exhibited higher expansion over multiple rounds of antigen exposure compared with cells grown with IL-7 + 15 alone (**Figure 5B**), a pattern consistently observed across all independent donors analyzed (n = 4) from day 4 onward. As expected, repeated stimulation increased T_TE_ frequency in both conditions, with no significant differences in memory subset distribution at the end of the assay (after four antigen stimulations) (**Supplementary Figure 8A**).

We also evaluated the expression of activation and exhaustion markers at the assay endpoint (**Supplementary Figure 8B**). No differences were observed in the expression of CTLA-4, PD-1, LAG-3, or TIM-3. CART84 cells expanded with IL-21 showed a slight reduction in CD69 expression across all independent donors analyzed (n = 4), consistent with prior observations (**Figure 2A**).

### 3.6 Influence of IL-21 on CART84 function in long-term *in vitro* tumor rechallenge assays

**Figure 6:**
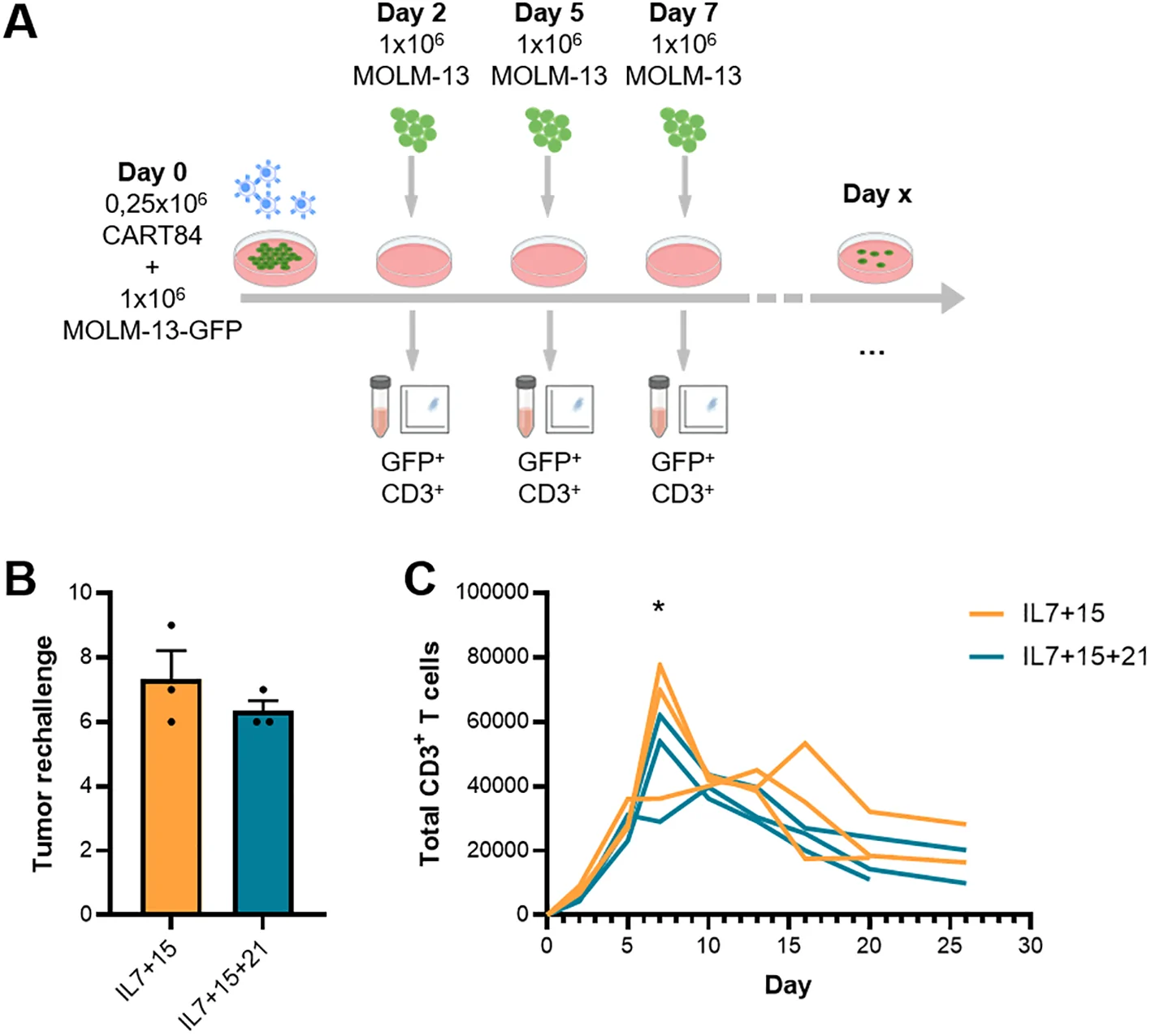
Long-term in vitro tumor rechallenge assay of CART84 expanded under different cytokine conditions. **(A)** Experimental design. **(B)** Long-term cytotoxicity against MOLM-13 cells. Bars represent the number of tumor rechallenges successfully controlled before loss of cytotoxic function. Statistical analysis was performed using a paired t test. **(C)** CD3^+^ cell counts at the indicated time points. After each measurement, CART84 were rechallenged with non-irradiated MOLM-13 cells at an initial effector-to-target ratio of 1:4. Statistical analysis was performed using mixed-effects models with donor-matched cytokine conditions (IL-7 + 15 and IL-7 + 15 + 21) and time points, followed by Sídák’s post-test for multiple comparisons. Data represent 3 independent donors (n = 3; same donors across all panels), mean ± S.E.M. *: *p* < 0.05. Comparisons not reaching significance are not shown.

To further evaluate CART84 function, CART84 cells expanded with IL-7 + 15, with or without IL-21 supplementation, were subjected to long-term *in vitro* tumor rechallenge assays. Non-irradiated MOLM-13 cells (E:T ratio of 1:4) were serially added following tumor clearance for as long as CART84 cells maintained tumor control (**Figure 6A**).

While IL-21 enhanced CART84 expansion during repeated stimulation with irradiated MOLM-13 cells, this advantage was not observed in the long-term serial tumor rechallenge assay. IL-21 did not improve the ability of CART84 cells to withstand successive tumor rechallenges (**Figure 6B**). Additionally, CART84 cells expanded in IL-7 + 15 reached higher peak expansion levels than IL-21-supplemented CART84 cells (**Figure 6C**).

Therefore, although IL-21-expanded CART84 cells displayed superior proliferative responses under repeated stimulation with irradiated targets, this advantage did not translate into improved tumor control when challenged with non-irradiated MOLM-13 cells at a higher tumor burden (E:T ratio of 1:4) in the long-term *in vitro* rechallenge assay.

### 3.7 IL-21 does not enhance *in vivo* efficacy of CART84

**Figure 7:**
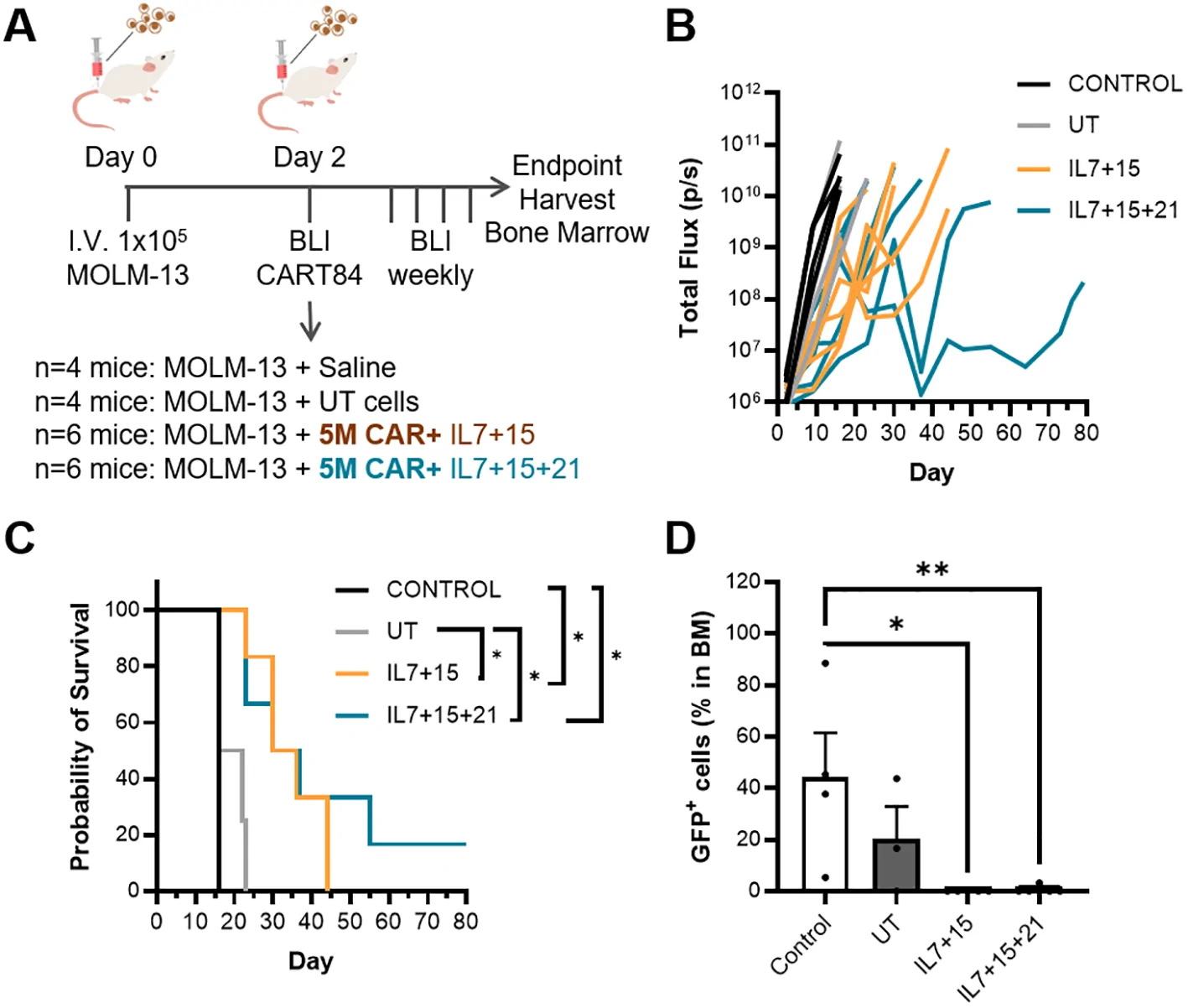
Effect of IL-21 addition on the in vivo efficacy of CART84. **(A)** Experimental design: on day 0, 1×10^5^ MOLM-13-GFP-ffLuc cells were administered intravenously (i.v.) to immunodeficient NSG female mice. On day 2, mice were allocated to experimental groups based on tumor burden to ensure similar baseline tumor loads. Subsequently, 5x10^6^ fresh CAR^+^ CART84 cells expanded with IL-7 + 15 or IL-7 + 15 + 21, untransduced (UT) cells, or saline were administered i.v. Leukemia burden was monitored weekly by bioluminescence imaging (BLI). Mice were euthanized upon reaching humane endpoint criteria or at experimental endpoint, and bone marrow was collected at sacrifice for flow cytometry analysis. The number of mice per group is indicated in the experimental design. All UT and CART84 cells were generated from a single healthy donor. **(B)** Leukemia burden was quantified by bioluminescence imaging (BLI; total flux, photons/s). Statistical analysis was performed using mixed-effects models matching by time point, followed by Tukey’s post-test for multiple comparisons. Comparisons not reaching significance are not shown. **(C)** Kaplan–Meier survival curves. Statistical analysis was performed using log-rank (Mantel–Cox) test with p-value correction for five comparisons (UT vs. IL-7 + 15, UT vs IL-7 + 15 + 21, Control vs IL-7 + 15, Control vs IL-7 + 15 + 21 and IL-7 + 15 vs IL-7 + 15 + 21). *: *p* < 0.01. **(D)** Frequency (%) of GFP^+^ tumor cells in the bone marrow (BM) at endpoint (following humane endpoint criteria or experimental endpoint). Statistical analysis was performed using a one-way ANOVA, followed by Tukey’s post-test for multiple comparisons. Data represent n = 4 mice for control, n = 3 mice for UT, n = 5 mice for IL-7 + 15, and n = 6 mice for IL-7 + 15 + 21, mean ± S.E.M. *: *p* < 0.05, **: *p* < 0.01. Comparisons not reaching significance are not shown.

The impact of IL-21 supplementation during CART84 expansion on *in vivo* efficacy was evaluated in two independent xenograft experiments. Immunodeficient NSG mice received 1x10^5^ MOLM-13 cells via tail vein and, two days later, were treated with an intravenous injection of 5x10^6^ CAR^+^ CART84 cells expanded in IL-7 + 15, with or without IL-21. Leukemia progression was monitored over time by bioluminescence imaging (**Figure 7A**; **Supplementary Figure 9A**). IL-21 supplementation during CART84 expansion did not confer a detectable advantage *in vivo*, as tumor progression kinetics were comparable between groups (**Figure 7B**; **Supplementary Figure 9B**). Survival was also similar in mice treated with CART84 expanded with IL-7 + 15 alone versus IL-7 + 15 plus IL-21 (**Figure 7C**; **Supplementary Figure 9C**). CART84 treatment significantly reduced disease burden irrespective of IL-21 addition, with a lower frequency of GFP^+^ tumor cells detected in bone marrow at the study endpoint (**Figure 7D**; **Supplementary Figure 9D**). The presence of CART84 in the bone marrow at endpoint was estimated by quantifying human CD45^+^CD3^+^ cells (HuCD45^+^HuCD3^+^), revealing comparable CART84 frequencies between IL-7 + 15– and IL-7 + 15 + 21–expanded CART84 cells (**Supplementary Figures 9E**, **10**).

Taken together, IL-21 supplementation during CART84 expansion did not enhance antitumor activity *in vivo* or increase CART84 cell frequencies in the bone marrow at the study endpoint in this model, despite its positive effect on CART84 expansion during repeated antigen stimulation *in vitro*.

### 3.8 IL-21 supplementation in a G-Rex-based expansion protocol

**Figure 8:**
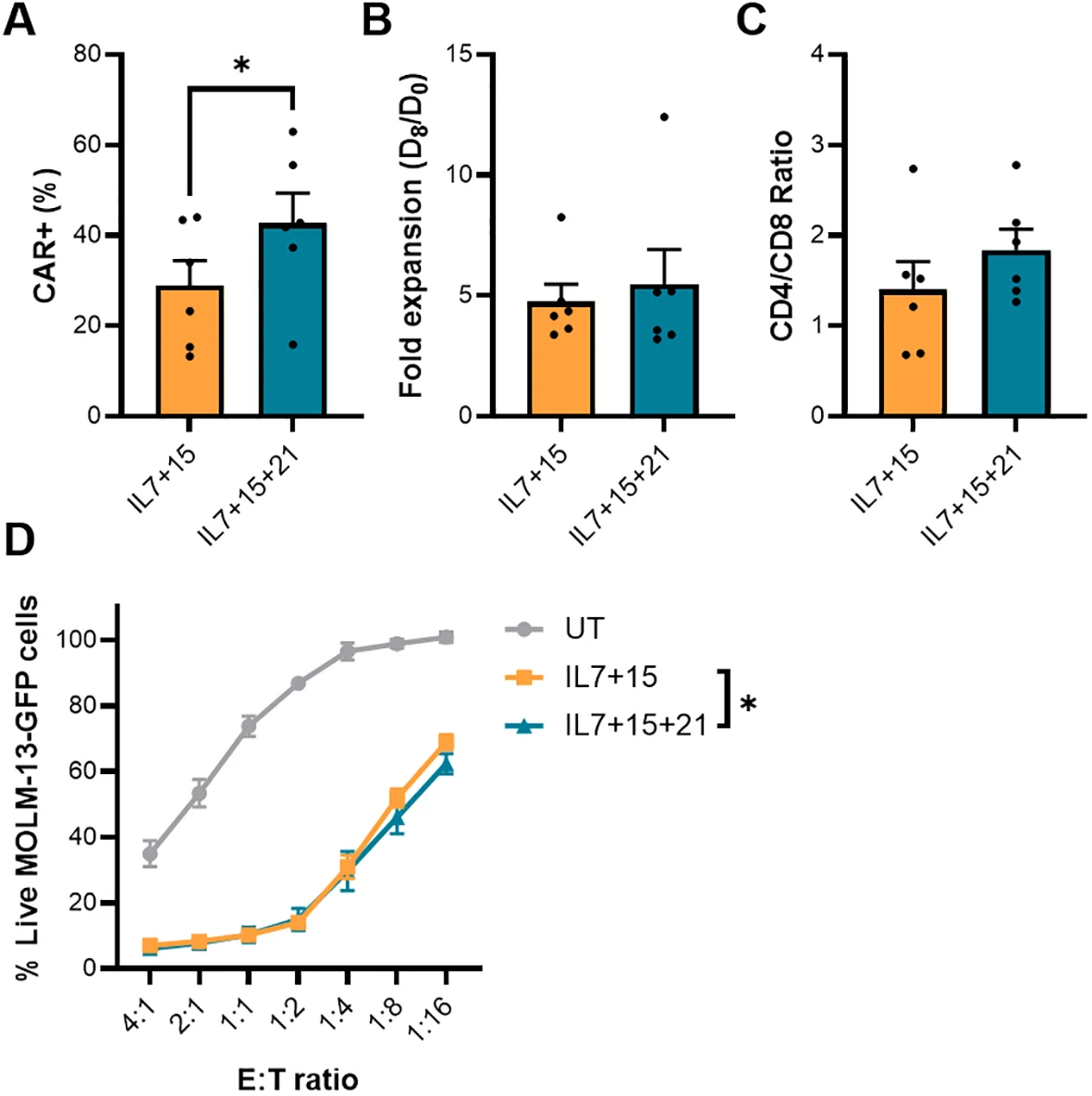
Characterization of G-Rex-expanded CART84 cells with or without IL-21. **(A)** Percentage of CAR^+^ cells. **(B)** Fold expansion. **(C)** CD4/CD8 ratio. Statistical analysis was performed with a paired t test. **(D)** Untransduced (UT) T cells and CART84 were co-cultured with MOLM-13 for 24 h at the indicated effector-to-target (E:T) ratios. The percentage of CAR^+^ cells was normalized to the lowest CAR expression for each donor using UT cells. Statistical analysis was performed using two-way ANOVA with donor-matched cytokine conditions (IL-7 + 15 and IL-7 + 15 + 21), followed by Sídák’s post-test for multiple comparisons. Data represent 6 independent donors (n = 6; same donors across all panels), mean ± S.E.M. *: *p* < 0.05. Comparisons not reaching significance are not shown.

To evaluate IL-21 supplementation in a GMP-scalable manufacturing setting, healthy donor T cells were activated, transduced, and expanded using a Gas-Permeable Rapid Expansion (G-Rex)-based protocol in the presence of IL-7 + 15 with or without IL-21. In contrast to conventional expansion conditions, IL-21 supplementation in the G-Rex system was associated with increased CAR expression across all independent donors analyzed (n = 6) (**Figure 8A**). No significant differences were observed in fold expansion or CD4/CD8 composition compared with IL-7 + 15 alone (**Figures 8B, C**).

To assess whether IL-21 induced functional changes, cytotoxicity assays against MOLM-13 cells were performed after normalizing effector CAR^+^ cells to the lowest CAR expression level within each donor using untransduced (UT) cells. In this setting, IL-21 expanded CART84 showed slightly improved killing of MOLM-13 cells, which was only evident at lower effector-to-target ratios (**Figure 8D**).

In G-Rex-expanded CART84 cells, IL-21 supplementation was associated with a moderate but statistically significant increase in CD4^+^ T_CM_ cells (**Supplementary Figure 11**), accompanied by a reduction in T_EM_ and T_TE_ CD4^+^ populations, a pattern consistently observed across all independent donors (n = 6). Although IL-21 supplementation did not increase T_CM_ frequencies under non–G-Rex expansions compared to IL-7 + 15 alone (**Figure 1D**), the overall trend toward reduced representation of more differentiated T-cell subsets was consistent across both experimental settings. In contrast to conventional CART84 expansion (**Figure 1D**), IL-21 in the G-Rex system slightly increased the frequency of T_CM_ cells within the CD8^+^ compartment (**Supplementary Figure 11**).

Regarding activation and exhaustion marker expression (**Supplementary Figure 12**), an increase in the frequency of LAG-3^+^ cells, particularly within the CD8^+^ compartment, remained the most prominent effect associated with IL-21 supplementation, similar to the pattern observed under conventional expansion (**Figure 2A**). This effect was consistently observed across all independent donors analyzed (n = 6). However, unlike conventionally-expanded CART84 cells, IL-21 supplementation in the G-Rex system was associated with minor changes in TIM-3 patterns, including a slight increase in TIM-3^+^ CD4^+^ cell frequencies and a reduction in TIM-3^+^ CD8^+^ cell frequencies; as well as a modest reduction in PD-1^+^ CD8^+^ T cells compared to IL7 + 15. In addition, IL-21 induced a small reduction in double-positive PD-1^+^TIM-3^+^ cells only in the CD8^+^ compartment, whereas no differences were detected in PD-1^+^LAG-3^+^ double-positive populations.

Overall, assessment of IL-21 supplementation in a G-Rex-based expansion protocol, performed to better approximate clinically relevant GMP manufacturing conditions, showed an increase in CAR expression together with a slight enrichment of less differentiated T-cell subsets. However, these changes were not associated with improved expansion and were accompanied by only limited effects on cytotoxic activity.

### 3.9 Validation of IL-21 effects in AML patient-derived CART84 cells

To evaluate whether IL-21 could improve the fitness of T cells from AML patients, which frequently exhibit features of dysfunction and exhaustion due to prior treatments and the immunosuppressive AML microenvironment (45, 46), CART84 cells were generated from AML patient-derived T cells under conventional expansion conditions with IL-7 + 15 ± IL-21. Patient and disease characteristics are summarized in **Supplementary Table 1**.

**Figure 9:**
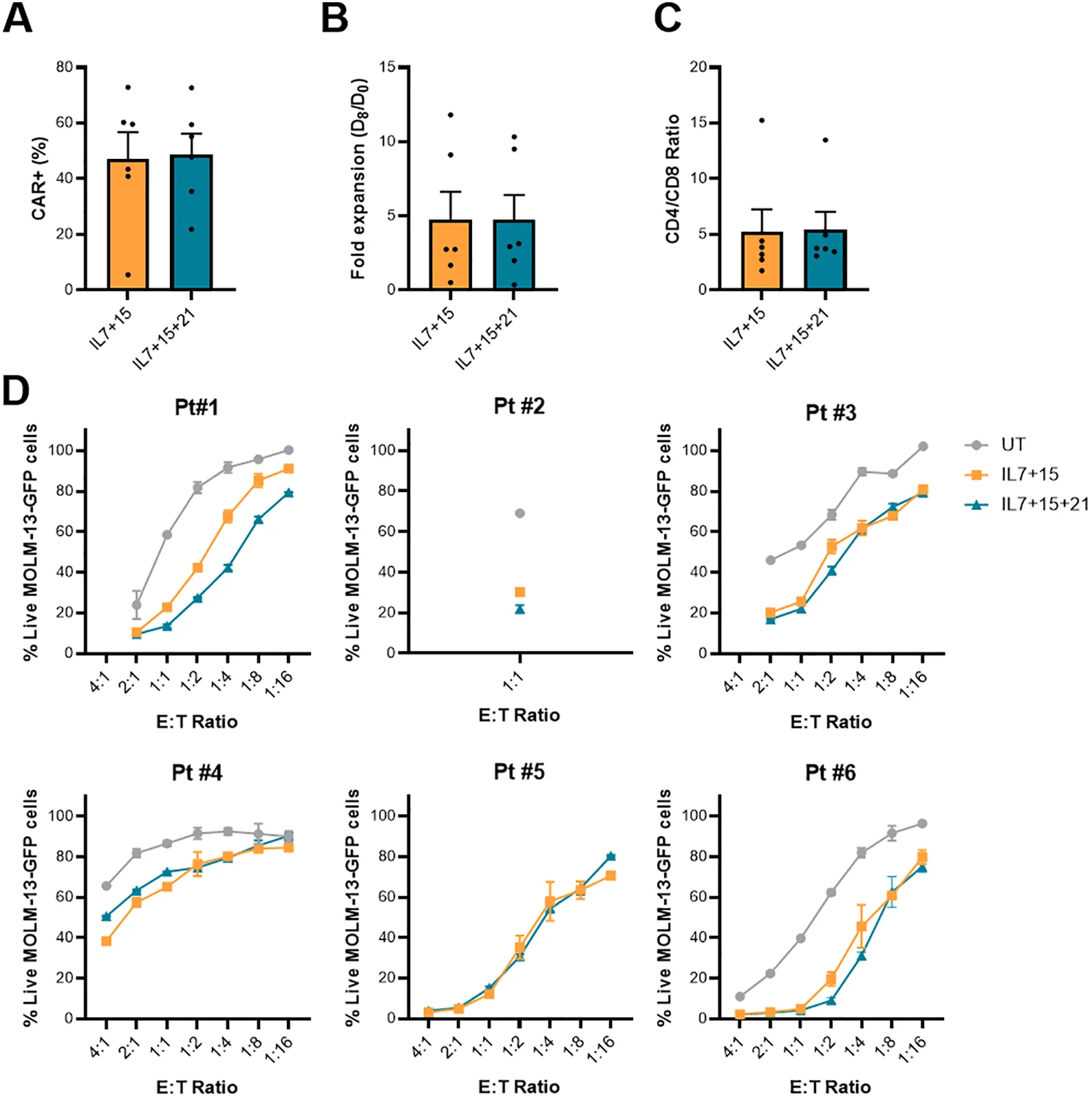
Characterization of CART84 cells generated from AML patient-derived T cells with or without IL-21. **(A)** Percentage of CAR^+^ cells. **(B)** Fold expansion (non-cryopreserved samples were expanded for 8 days, whereas cryopreserved samples were expanded for 9 days). **(C)** CD4/CD8 ratio. Statistical analysis was performed with a paired t test. Data represent 6 independent AML patient samples (n = 6), mean ± S.E.M. Comparisons not reaching significance are not shown. **(D)** Untransduced (UT) cells and CART84 generated under the indicated cytokine conditions were co-cultured with MOLM-13-GFP-ffLuc cells for 24 h at the indicated effector-to-target (E:T) ratios. Each graph represents an individual AML patient sample; due to inter-patient heterogeneity, cytotoxicity data were not pooled and no statistical analysis was performed. Owing to limited sample availability, CART84 cells from patient #1 could only be tested at E:T ratios ≤ 2:1, patient #2 could only be evaluated at a 1:1 E:T ratio, and UT cells could not be generated for patient #5. Data represent 6 independent patients (n = 6), showing 2 technical duplicates for each patient, mean ± S.E.M. Pt: patient.

CART84 cells were successfully generated from all patient samples and displayed antileukemic activity against MOLM-13 cells (**Figure 9**). However, IL-21 supplementation did not affect CAR expression (**Figure 9A**), fold expansion (**Figure 9B**), or CD4/CD8 composition (**Figure 9C**). Likewise, although cytotoxic activity varied between patient-derived products, IL-21 did not consistently enhance MOLM-13 killing across donors (**Figure 9D**).

To determine whether IL-21 influenced the phenotype of AML patient-derived CART84 cells before and after antigen encounter, T-cell memory subsets and activation/exhaustion marker expression were analyzed both at the end of CART84 expansion (baseline) and after the cytotoxicity assay against MOLM-13 cells (post-assay) (**Supplementary Figure 13**). IL-21 did not alter the distribution of T-cell memory subsets at the end of CART84 expansion. Analysis of activation- and exhaustion-associated markers revealed limited differences, including lower frequencies of PD-1^+^ and PD-1^+^TIM-3^+^ cells together with higher frequencies of LAG-3^+^ cells within the CD8^+^ compartment of IL-21-expanded CART84 cells (**Supplementary Figure 13**).

Following the cytotoxicity assay, T-cell memory subset distribution remained comparable between cytokine conditions. In CD4^+^ cells, PD-1^+^ and PD-1^+^TIM-3^+^ cell frequencies increased in the IL-7 + 15 condition, resulting in comparatively lower post-assay frequencies in the IL-7 + 15 + 21 condition. In the CD8^+^ compartment, LAG-3^+^ frequencies decreased post-assay in both cytokine conditions, whereas lower frequencies of PD-1^+^TIM-3^+^ cells were maintained in IL-21-expanded CD8^+^ CART84 cells post-assay (**Supplementary Figure 13**). Taken together, IL-21 supplementation was associated with limited differences in activation- and exhaustion-associated marker expression before and after antigen exposure, while T-cell memory subset distribution remained unchanged.

Overall, CART84 cells were successfully generated from AML patient-derived T cells and retained antileukemic activity. However, IL-21 supplementation did not confer a clear advantage in terms of expansion, cytotoxic activity, or T-cell memory phenotype.

## 4 Discussion

CAR T-cell manufacturing can influence the differentiation state and metabolic fitness of the final product, with direct implications for potency, expansion, and persistence (2, 6, 8, 9, 16). Here, we investigated whether IL-21 supplementation during IL-7 + 15 expansion could further improve CAR T cells for the treatment of AML, using IL-7 + 15 alone as the primary comparator and IL-2 as a preclinical benchmark. In healthy donor-derived CART84 cells, IL-21 supplementation was linked to an enrichment of less differentiated T-cell subsets, enhanced expansion following repeated antigen stimulation, and increased CAR expression under G-Rex-based expansion conditions. However, these effects were not accompanied by consistent improvements in cytotoxicity, sustained tumor control upon serial rechallenge, or *in vivo* efficacy in a MOLM-13 AML model. In AML patient-derived CART84 cells, IL-21 did not improve CAR expression, expansion, memory subset distribution, or consistently enhance cytotoxic activity. Collectively, IL-21 supplementation was associated with measurable phenotypic changes in CART84 cells, but these did not translate into a clear functional advantage over IL-7 + 15 alone.

Multi-omics and high-throughput approaches are increasingly used to identify determinants of CAR T-cell efficacy and persistence (13, 14, 58, 61). Transcriptional signatures associated with early memory subsets have been linked to improved persistence (13, 14), and products enriched for these features have shown enhanced efficacy in preclinical (20, 37, 62) and clinical studies (6–12). Most evidence derives from B−cell malignancies. In AML, the role of product fitness—such as stem-like programs—remains less well-defined. Strategies to enhance CAR T-cell fitness include selecting favorable T-cell subsets (10, 17–20), reducing tonic signaling (63, 64), or engineering CARs to secrete persistence-supporting cytokines (e.g. armored CAR T-cells or TRUCKs) (65, 66).

Cytokines used during ex vivo expansion are key determinants of CAR T-cell differentiation phenotypes (16, 21, 22). High-dose IL-2, used in some FDA-approved processes (22), supports robust proliferation but promotes effector differentiation (23, 24). IL-7 and IL-15 better preserve memory phenotypes while enabling efficient expansion (10, 24, 54). In preclinical CAR T-cell models, IL-21 has been associated with reduced terminal differentiation and enhanced proliferative capacity (17, 32, 35–37, 40), primarily via STAT1/STAT3 signaling, in contrast to other γ-chain cytokines that activate STAT5 (27). STAT3 programs promote memory-associated gene expression and stemness (e.g., Tcf7) (13, 67), providing a mechanistic rationale for exploring IL-21 as a strategy to improve CAR T-cell fitness.

Beyond soluble supplementation, IL-21 has been incorporated into TRUCKs, either constitutively (34, 38, 41, 42, 68) or inducibly (39), enhancing antitumor activity. IL-15 and IL-21-secreting CAR T-cells targeting GPC3 are under clinical evaluation (NCT02932956 and NCT02905188) (38). However, whether IL-21 confers clinical advantage remains uncertain. Thus, the incorporation of IL-21 as a GMP-compatible media supplement motivated our evaluation, as it could enhance cell yield or fitness without extending manufacturing timelines or substantially increasing costs (43, 44).

Previous studies have linked IL-21 supplementation to enhanced lentiviral transduction efficiency, CAR T-cell expansion, and enrichment of less differentiated CAR T-cell subsets, such as T_SCM_ (17, 35–37, 39, 40). However, most studies reporting increased T_SCM_ with IL-21 included additional variables (e.g., reduced stimulation, naïve precursor enrichment, or stemness-supporting supplements) (17, 20, 36, 37), or combined IL-21 supplementation with alternative CAR engineering approaches (35, 39). In our study, the effects of IL-21 varied according to the expansion platform and donor source. CAR expression was increased only in G-Rex-expanded CART84 cells, whereas IL-21 did not provide an expansion benefit over IL-7 + 15 alone. Moreover, IL-21 did not induce T_SCM_ enrichment as previously reported (32, 35, 36). Instead, IL-21 was associated with a slight increase in CD4^+^ T_CM_ frequency and a reduction in more differentiated subsets in healthy donor-derived CART84 cells. Although IL-21 effects have predominantly been described in CD8^+^ T cells (26, 28, 31, 67), the phenotypic changes observed here were more evident within the CD4^+^ compartment. Notably, IL-21 did not improve memory subset distribution in AML patient-derived CART84 cells. The advanced age of the patients (67 ± 16 years old; mean ± S.D.), and the known accumulation of differentiated T-cell populations with age (69) may have contributed to this observation.

Beyond effects on T-cell differentiation, IL-21 also modulated activation and exhaustion marker expression in CART84 cells, with the most pronounced and consistent effect being increased LAG-3 expression in CD8^+^ T cells. Similar observations have been reported in activated murine T cells (31), and strategies limiting LAG-3 signaling in IL-21-secreting TRUCKs are being explored (70). LAG-3 can be transiently induced during physiological T-cell activation (70), and is also upregulated by other γ-chain cytokines (71, 72), but the mechanism mediated by IL-21 remains unclear. Therefore, our findings may reflect more sustained LAG-3 expression following activation in the presence of IL-21, rather than a dysfunctional state. Further studies may help clarify the mechanistic relationship between IL-21 supplementation and LAG-3 expression.

IL-21 was also associated with slight increase in basal mitochondrial ATP production relative to IL-2, in line with previous reports (26, 30–32). However, no significant metabolic differences were observed compared with IL-7 + 15 alone. These findings suggest a limited effect of IL-21 on CART84 metabolic programming under our experimental conditions. Studies reporting enhanced respiratory reserve in memory-enriched T-cell products often evaluated cells after longer culture durations (17, 59), which may partly explain the absence of SRC differences in our study.

The phenotypic and metabolic changes associated with IL-21 supplementation did not consistently translate into improved CART84 function, suggesting that these features alone may be insufficient to predict enhanced CART84 activity. CART84 expanded in IL-7 + 15 (with or without IL-21) showed superior killing only against MOLM-13 compared with IL-2, without differences against other target cell lines. The preferential activity observed against MOLM-13 cells may therefore reflect target cell-specific characteristics rather than differences in CART84 fitness. IL-21 supplementation was associated with a slight increase in MOLM-13 killing in G-Rex-expanded CART84 cells, but this effect was minimal, restricted to low effector-to-target ratios, and not consistently reproduced in AML patient-derived CART84. Du and collaborators reported that IL-21 addition enhanced cytotoxic activity of HER2-directed CAR T-cells against solid tumor cell lines, associated with increased IFNγ and granzyme B secretion (40). In the present study, IL-21 did not significantly increase IFNγ and granzyme B production, consistent with its limited impact on cytotoxic activity.

During repeated antigen stimulation, IL-21-expanded CART84 cells showed enhanced expansion following successive rounds of antigen exposure. However, this proliferative advantage was not reproduced in the long-term *in vitro* rechallenge assay, where tumor control remained comparable between cytokine conditions. Similarly, IL-21 supplementation did not improve efficacy or CART84 persistence in the MOLM-13 xenograft model, as leukemia progression and survival were comparable between groups. Although the rapid disease kinetics in this model may have limited the detection of subtle effects, the phenotypic and proliferative changes associated with IL-21 supplementation in healthy donor-derived CART84 cells did not consistently translate into improvements in cytotoxicity *in vitro* or tumor control *in vivo*. This suggests that CART84 features modulated by IL-21 are not the primary determinants of antitumor efficacy in the AML models evaluated here. Rather, effective antitumor responses may depend more strongly on immediate cytotoxic function and the ability to operate under inhibitory conditions. These findings are in line with observations that CAR T-cell phenotypic features do not always predict clinical outcomes in AML (73). IL-21-secreting TRUCKs have demonstrated enhanced antitumor activity in preclinical settings (34, 38, 39, 42), suggesting that sustained IL-21 signaling may have different functional consequences than transient exposure during ex vivo CAR T-cell expansion.

Patient-derived T cells can differ substantially from healthy donor T cells in phenotype, functional fitness, and manufacturability, particularly in AML, where chronic immune dysfunction may impair antitumor responses (45, 46). We therefore considered AML patient-derived T cells to be the most clinically relevant setting in which to evaluate the potential benefits of IL-21 supplementation. However, no clear improvement was observed in CAR expression, expansion, memory subset distribution, or cytotoxic activity, suggesting that IL-21 supplementation alone is insufficient to substantially improve AML patient-derived CART84 products.

Several aspects of this study should be considered when interpreting the findings. *In vivo* efficacy was evaluated using a rapidly progressing MOLM-13 xenograft model, which may have limited the detection of subtle differences in CART84 antitumor activity. While this model is widely used for preclinical evaluation of AML-directed CAR T cells, additional *in vivo* models may help further assess the impact of IL-21 supplementation across different disease settings. In addition, the study was designed primarily to evaluate whether the phenotypic effects previously associated with IL-21 supplementation during CAR T-cell manufacturing could be reproduced in CART84 cells and translate into improved functional properties. However, the molecular mechanisms underlying the observed effects were not comprehensively investigated.

In conclusion, IL-21 supplementation was associated with enrichment of less differentiated T-cell subsets in healthy donor-derived CART84 cells. However, these effects were not accompanied by consistent improvements in cytotoxicity or *in vivo* efficacy. Moreover, IL-21 addition did not confer a clear advantage in expansion, cytotoxic activity, or T-cell memory phenotype in AML patient-derived CART84 cells. Overall, our results do not warrant routine incorporation of IL-21 into IL-7 + 15-based CART84 manufacturing protocols and support the need to evaluate cytokine supplementation strategies within the context of each CAR construct and disease setting.

## Data Availability

The original contributions presented in the study are included in the article/**Supplementary Material**. Further inquiries can be directed to the corresponding authors.

## References

[1] Qin H Zhou Z Shi R Mai Y Xu Y Peng F . Insights into next-generation immunotherapy designs and tools: molecular mechanisms and therapeutic prospects. J Hematol Oncol. (2025) 18. doi: 10.1186/s13045-025-01701-6 40483473 PMC12145627

[2] Tao Z Chyra Z Kotulová J Celichowski P Mihályová J Charvátová S . Impact of T cell characteristics on CAR-T cell therapy in hematological Malignancies. Blood Cancer J. (2024) 14:213. doi: 10.1038/s41408-024-01193-6 39627220 PMC11615218

[3] Gattinoni L Lugli E Ji Y Pos Z Paulos CM Quigley MF . A human memory T cell subset with stem cell-like properties. Nat Med. (2011) 17:1290–7. doi: 10.1038/nm.2446 21926977 PMC3192229

[4] Lugli E Dominguez MH Gattinoni L Chattopadhyay PK Bolton DL Song K . Superior T memory stem cell persistence supports long-lived T cell memory. J Clin Invest. (2013) 123:594–9. doi: 10.1172/JCI66327 23281401 PMC3561805

[5] Zhan W Lai L Ran S Li Y Zhao J Wu J . Stem-like T cells: molecular regulation, functional diversity, and therapeutic implications across diseases. J Transl Med. (2025) 23. doi: 10.1186/s12967-025-07383-5 41340063 PMC12676829

[6] Fraietta JA Lacey SF Orlando EJ Pruteanu-Malinici I Gohil M Lundh S . Determinants of response and resistance to CD19 chimeric antigen receptor (CAR) T cell therapy of chronic lymphocytic leukemia. Nat Med. (2018) 24:563–71. doi: 10.1038/s41591-018-0010-1 29713085 PMC6117613

[7] Aldoss I Khaled SK Wang X Palmer J Wang Y Wagner JR . Favorable activity and safety profile of memory-enriched CD19-targeted chimeric antigen receptor T-cell therapy in adults with high-risk relapsed/refractory ALL. Clin Cancer Res. (2023) 29:742–53. doi: 10.1158/1078-0432.CCR-22-2038 36255386 PMC10544259

[8] Deng Q Han G Puebla-Osorio N Ma MCJ Strati P Chasen B . Characteristics of anti-CD19 CAR T cell infusion products associated with efficacy and toxicity in patients with large B cell lymphomas. Nat Med. (2020) 26:1878–87. doi: 10.1038/s41591-020-1061-7 33020644 PMC8446909

[9] Filosto S Vardhanabhuti S Canales MA Poiré X Lekakis LJ de Vos S . Product attributes of CAR T-cell therapy differentially associate with efficacy and toxicity in second-line large B-cell lymphoma (ZUMA-7). Blood Cancer Discov. (2024) 5:21–33. doi: 10.1158/2643-3230.BCD-23-0112 37983485 PMC10772511

[10] Xu Y Zhang M Ramos CA Durett A Liu E Dakhova O . Closely related T-memory stem cells correlate with *in vivo* expansion of CAR.CD19-T cells and are preserved by IL-7 and IL-15. Blood. (2014) 123:3750–9. doi: 10.1182/blood-2014-01-552174 24782509 PMC4055922

[11] Lin Y Raje NS Berdeja JG Siegel DS Jagannath S Madduri D . Idecabtagene vicleucel for relapsed and refractory multiple myeloma: post hoc 18-month follow-up of a phase 1 trial. Nat Med. (2023) 29:2286–94. doi: 10.1038/s41591-023-02496-0 37592106 PMC10504071

[12] Štach M Pytlík R Šmilauerová K Rychlá J Mucha M Musil J . Characterization of the input material quality for the production of tisagenlecleucel by multiparameter flow cytometry and its relation to the clinical outcome. Pathol Oncol Res. (2023) 29. doi: 10.3389/pore.2023.1610914 37151356 PMC10156917

[13] Chen GM Chen C Das RK Gao P Chen CH Bandyopadhyay S . Integrative bulk and single-cell profiling of premanufacture T-cell populations reveals factors mediating long-term persistence of CAR T-cell therapy. Cancer Discov. (2021) 11:2186–99. doi: 10.1158/2159-8290.CD-20-1677 33820778 PMC8419030

[14] Anderson ND Birch J Accogli T Criado I Khabirova E Parks C . Transcriptional signatures associated with persisting CD19 CAR-T cells in children with leukemia. Nat Med. (2023) 29:1700–9. doi: 10.1038/s41591-023-02415-3 37407840 PMC10353931

[15] Arcangeli S Bove C Mezzanotte C Camisa B Falcone L Manfredi F . CAR T cell manufacturing from naive/stem memory T lymphocytes enhances antitumor responses while curtailing cytokine release syndrome. J Clin Invest. (2022) 132. doi: 10.1172/JCI150807 35503659 PMC9197529

[16] Wang X Liao Y Liu D Zheng J Shi M . Presetting CAR-T cells during ex vivo biomanufacturing. Mol Ther. (2025) 33:1380–406. doi: 10.1016/j.ymthe.2025.02.031 39988874 PMC11997485

[17] Sabatino M Hu J Sommariva M Gautam S Fellowes V Hocker JD . Generation of clinical-grade CD19-specific CAR-modified CD8 1 memory stem cells for the treatment of human B-cell Malignancies. Blood. (2016) 128:519–28. doi: 10.1182/blood-2015-11-683847 27226436 PMC4965906

[18] Turtle CJ Hanafi LA Berger C Gooley TA Cherian S Hudecek M . CD19 CAR-T cells of defined CD4+:CD8+ composition in adult B cell ALL patients. J Clin Invest. (2016) 126:2123–38. doi: 10.1172/JCI85309 27111235 PMC4887159

[19] Wang X Popplewell LL Wagner JR Naranjo A Blanchard MS Mott MR . Phase 1 studies of central memory-derived CD19 CAR T-cell therapy following autologous HSCT in patients with B-cell NHL. Blood. (2016) 17:2980–90. doi: 10.1182/blood-2015-12-686725 27118452 PMC4911862

[20] Meyran D Zhu JJ Butler J Tantalo D MacDonald S Nguyen TN . TSTEM-like CAR-T cells exhibit improved persistence and tumor control compared with conventional CAR-T cells in preclinical models. Sci Transl Med. (2023) 15. doi: 10.1126/scitranslmed.abk1900 37018415

[21] Dwyer CJ Knochelmann HM Smith AS Wyatt MM Rangel Rivera GO Arhontoulis DC . Fueling cancer immunotherapy with common gamma chain cytokines. Front Immunol. (2019) 10. doi: 10.3389/fimmu.2019.00263 30842774 PMC6391336

[22] Watanabe N Mo F McKenna MK . Impact of manufacturing procedures on CAR T cell functionality. Front Immunol. (2022) 13:876339. doi: 10.3389/fimmu.2022.876339 35493513 PMC9043864

[23] Chinen T Kannan AK Levine AG Fan X Klein U Zheng Y . An essential role for the IL-2 receptor in Treg cell function. Nat Immunol. (2016) 17:1322–33. doi: 10.1038/NI.3540 27595233 PMC5071159

[24] Zhou J Jin L Wang F Zhang Y Liu B Zhao T . Chimeric antigen receptor T (CAR-T) cells expanded with IL-7/IL-15 mediate superior antitumor effects. Protein Cell. (2019) 10:764–9. doi: 10.1007/s13238-019-0643-y 31250350 PMC6776495

[25] Feng B Bai Z Zhou X Zhao Y Xie YQ Huang X . The type 2 cytokine Fc-IL-4 revitalizes exhausted CD8+ T cells against cancer. Nature. (2024) 634:712–20. doi: 10.1038/s41586-024-07962-4 39322665 PMC11485240

[26] Li Y Bleakley M Yee C . IL-21 influences the frequency, phenotype, and affinity of the antigen-specific CD8 T cell response 1. J Immunol. (2005) 175:2261–9. doi: 10.4049/jimmunol.175.4.2261 16081794

[27] Zeng R Spolski R Casas E Zhu W Levy DE Leonard WJ . The molecular basis of IL-21-mediated proliferation. Blood. (2007) 109:4135–42. doi: 10.1182/blood-2006-10-054973 17234735 PMC1885510

[28] Hinrichs CS Spolski R Paulos CM Gattinoni L Kerstann KW Palmer DC . IL-2 and IL-21 confer opposing differentiation programs to CD8+ T cells for adoptive immunotherapy. Blood. (2008) 111:5326–33. doi: 10.1182/blood-2007-09-113050 18276844 PMC2396726

[29] Santegoets SJAM Turksma AW Suhoski MM Stam AG Albelda SM Hooijberg E . IL-21 promotes the expansion of CD27+ CD28+ tumor infiltrating lymphocytes with high cytotoxic potential and low collateral expansion of regulatory T cells. J Transl Med. (2013) 11. doi: 10.1186/1479-5876-11-37 23402380 PMC3626797

[30] Loschinski R Böttcher M Stoll A Bruns H Mackensen A Mougiakakos D . IL-21 modulates memory and exhaustion phenotype of T-cells in a fatty acid oxidation-dependent manner. Oncotarget. (2018) 9:13125–38. doi: 10.18632/oncotarget.24442 29568345 PMC5862566

[31] Hermans D Gautam S García-Cañaveras JC Gromer D Mitra S Spolski R . Lactate dehydrogenase inhibition synergizes with IL-21 to promote CD8+ T cell stemness and antitumor immunity. PNAS. (2020) 117:6047–55. doi: 10.1073/PNAS.1920413117 32123114 PMC7084161

[32] Zhang M Kong J Yin F Shi J Li J Qiu Z . Optimizing CAR-T cell culture: differential effects of IL-2, IL-12, and IL-21 on CAR-T cells. Cytokine. (2024) 184. doi: 10.1016/j.cyto.2024.156758 39299100

[33] Li Y Cong Y Jia M He Q Zhong H Zhao Y . Targeting IL-21 to tumor-reactive T cells enhances memory T cell responses and anti-PD-1 antibody therapy. Nat Commun. (2021) 12. doi: 10.1038/s41467-021-21241-0 33574265 PMC7878483

[34] Markley JC Sadelain M . IL-7 and IL-21 are superior to IL-2 and IL-15 in promoting human T cell-mediated rejection of systemic lymphoma in immunodeficient mice. Blood. (2010) 115:3508–19. doi: 10.1182/blood-2009-09-241398 20190192 PMC2867264

[35] Singh H Figliola MJ Dawson MJ Huls H Olivares S Switzer K . Reprogramming CD19-specific T cells with IL-21 signaling can improve adoptive immunotherapy of B-lineage Malignancies. Cancer Res. (2011) 71:3516–27. doi: 10.1158/0008-5472.CAN-10-3843 21558388 PMC3096697

[36] Alvarez-Fernández C Escribà-Garcia L Vidal S Sierra J Briones J . A short CD3/CD28 costimulation combined with IL-21 enhance the generation of human memory stem T cells for adoptive immunotherapy. J Transl Med. (2016) 14. doi: 10.1186/s12967-016-0973-y 27435312 PMC4952071

[37] Alvarez-Fernández C Escribà-Garcia L Caballero AC Escudero-López E Ujaldón-Miró C Montserrat-Torres R . Memory stem T cells modified with a redesigned CD30-chimeric antigen receptor show an enhanced antitumor effect in Hodgkin lymphoma. Clin Trans Immunol. (2021) 10. doi: 10.1002/cti2.1268 33968404 PMC8082716

[38] Batra SA Rathi P Guo L Courtney AN Fleurence J Balzeau J . Glypican-3-specific CAR T cells coexpressing IL15 and IL21 have superior expansion and antitumor activity against hepatocellular carcinoma. Cancer Immunol Res. (2020) 8:309–20. doi: 10.1158/2326-6066.CIR-19-0293 31953246 PMC10765595

[39] Štach M Ptáčková P Mucha M Musil J Klener P Otáhal P . Inducible secretion of IL-21 augments anti-tumor activity of piggyBac-manufactured chimeric antigen receptor T cells. Cytotherapy. (2020) 22:744–54. doi: 10.1016/j.jcyt.2020.08.005 32950390

[40] Du L Nai Y Shen M Li T Huang J Han X . IL-21 optimizes the CAR-T cell preparation through improving lentivirus mediated transfection efficiency of T cells and enhancing CAR-T cell cytotoxic activities. Front Mol Biosci. (2021) 8:675179. doi: 10.3389/fmolb.2021.675179 34179083 PMC8220804

[41] Chen S Gong F Liu S Xie Y Ye X Lin X . IL-21- and CXCL9-engineered GPC3-specific CAR-T cells combined with PD-1 blockade enhance cytotoxic activities against hepatocellular carcinoma. Clin Exp Med. (2024) 24:204. doi: 10.1007/s10238-024-01473-2 39196390 PMC11358300

[42] Han K Wang X Chen G Liu W Mei X Yang Y . Targeted therapy of multiple myeloma by IL21-NKG2D CAR-T cells. J Invest Med. (2025) 73:45–53. doi: 10.1177/10815589241291846 39373147

[43] Mucha M Štach M Kaštánková I Rychlá J Vydra J Lesný P . Good manufacturing practice-grade generation of CD19 and CD123-specific CAR-T cells using piggyBac transposon and allogeneic feeder cells in patients diagnosed with B-cell non-Hodgkin lymphoma and acute myeloid leukemia. Front Immunol. (2024) 15. doi: 10.3389/fimmu.2024.1415328 39192973 PMC11347927

[44] Caballero AC Ujaldón-Miró C Pujol-Fernández P Montserrat-Torres R Guardiola-Perello M Escudero-López E . HSP-CAR30 with a high proportion of less-differentiated T cells promotes durable responses in refractory CD30 + lymphoma. Blood. (2025) 16:1788–801. doi: 10.1182/blood.2024026758 39841453

[45] Epperly R Gottschalk S Velasquez MP . A bump in the road: how the hostile AML microenvironment affects CAR T cell therapy. Front Oncol. (2020) 10:517929. doi: 10.3389/fonc.2020.00262 32185132 PMC7058784

[46] Mardiana S Gill S . CAR T cells for acute myeloid leukemia: state of the art and future directions. Front Oncol. (2020) 10:535403. doi: 10.3389/fonc.2020.00697 32435621 PMC7218049

[47] Haubner S Subklewe M Sadelain M . Honing CAR T cells to tackle acute myeloid leukemia. Blood. (2025) 145:1113–25. doi: 10.1182/blood.2024024063 39630061

[48] Cuenca M Sintes J Lányi Á Engel P . CD84 cell surface signaling molecule: an emerging biomarker and target for cancer and autoimmune disorders. Clin Immunol. (2019) 204:43–9. doi: 10.1016/J.CLIM.2018.10.017 30522694

[49] Oporto Espuelas M Kanannejad S Michelozzi IM Gómez-Castañeda E Xenakis T Kirtsios E . Single cell multimodal framework identifies CD84 as a leukaemic stem cell target in paediatric AML. Blood. (2024) 144:4317. doi: 10.1182/blood-2024-204084

[50] Zhu Y Murtadha M Liu M Caserta E Napolitano O Nguyen LXT . Identification of CD84 as a potent survival factor in acute myeloid leukemia. J Clin Invest. (2025) 135. doi: 10.1172/JCI176818 40198133 PMC12126229

[51] Pérez-Amill L Armand-Ugón M Val-Casals M Martín-Herreros B Álamo JR Peña S . A novel chimeric antigen receptor T-cell therapy targeting CD84 for the treatment of acute myeloid and T-cell lymphoblastic leukemias. Leukemia. (2025) 39:2432–41. doi: 10.1038/s41375-025-02705-4 40770072 PMC12463657

[52] Pigazzi M Merlini S Da Ros A Marini O Faggin G Fortuna N . CD84 is a specific target for acute myeloid leukemia CAR-T cell therapy. Nat Commun. (2026) 13:e133-e143. doi: 10.1038/S41467-026-72361-4. Preprint. 42098093 PMC13369472

[53] Castella M Boronat A Martín-Ibáñez R Rodríguez V Suñé G Caballero M . Development of a novel anti-CD19 chimeric antigen receptor: a paradigm for an affordable CAR T cell production at academic institutions. Mol Ther Methods Clin Dev. (2019) 12:134–44. doi: 10.1016/j.omtm.2018.11.010 30623002 PMC6319086

[54] Castella M Caballero-Baños M Ortiz-Maldonado V González-Navarro EA Suñé G Antoñana-Vidósola A . Point-of-care CAR T-cell production (ARI-0001) using a closed semi-automatic bioreactor: experience from an academic phase i clinical trial. Front Immunol. (2020) 11. doi: 10.3389/fimmu.2020.00482 32528460 PMC7259426

[55] Ortiz-Maldonado V Rives S Castellà M Alonso-Saladrigues A Benítez-Ribas D Caballero-Baños M . CART19-BE-01: a multicenter trial of ARI-0001 cell therapy in patients with CD19+ relapsed/refractory Malignancies. Mol Ther. (2021) 29:636–44. doi: 10.1016/j.ymthe.2020.09.027 33010231 PMC7854276

[56] Ortiz-Maldonado V Martínez-Cibrian N Alserawan L Español-Rego M Navarro-Velázquez S Albiol N . Varnimcabtagene autoleucel for adults with relapsed or refractory B-cell precursor acute lymphoblastic leukaemia in Spain (CART19-BE-02): a multicentre, single-arm, phase 2 trial. Lancet Haematol. (2026) 17:6205. doi: 10.1016/S2352-3026(25)00328-X. Preprint. 41651004

[57] Oliver-Caldés A González-Calle V Cabañas V Español-Rego M Rodríguez-Otero P Reguera JL . Fractionated initial infusion and booster dose of ARI0002h, a humanised, BCMA-directed CAR T-cell therapy, for patients with relapsed or refractory multiple myeloma (CARTBCMA-HCB-01): a single-arm, multicentre, academic pilot study. Lancet Oncol. (2023) 24:913–24. doi: 10.1016/S1470-2045(23)00222-X 37414060

[58] Guerrero-Murillo M Rill-Hinarejos A Trincado JL Bataller A Ortiz-Maldonado V Benítez-Ribas D . Integrative single-cell multi-omics of CD19-CARpos and CARneg T cells suggest drivers of immunotherapy response in B cell neoplasias. Cell Rep Med. (2024) 5. doi: 10.1016/j.xcrm.2024.101803 39471818 PMC11604525

[59] Kawalekar OU O'Connor RS Fraietta JA Guo L McGettigan SE Posey AD Jr . Distinct signaling of coreceptors regulates specific metabolism pathways and impacts memory development in CAR T cells. Immunity. (2016) 44:380–90. doi: 10.1016/j.immuni.2016.01.021 26885860 PMC13498396

[60] Menk AV Scharping NE Moreci RS Zeng X Guy C Salvatore S . Early TCR signaling induces rapid aerobic glycolysis enabling distinct acute T cell effector functions. Cell Rep. (2018) 22:1509–21. doi: 10.1016/j.celrep.2018.01.040 29425506 PMC5973810

[61] Knudsen NH Escobar G Korell F Kienka T Nobrega C Anderson S . *In vivo* CRISPR screens identify modifiers of CAR T cell function in myeloma. Nature. (2025) 646:953–62. doi: 10.1038/s41586-025-09489-8 40993381 PMC12806180

[62] Sommermeyer D Hudecek M Kosasih PL Gogishvili T Maloney DG Turtle CJ . Chimeric antigen receptor-modified T cells derived from defined CD8+ and CD4+ subsets confer superior antitumor reactivity *in vivo*. Leukemia. (2015) 30:492–500. doi: 10.1038/leu.2015.247 26369987 PMC4746098

[63] Chen J Qiu S Li W Wang K Zhang Y Yang H . Tuning charge density of chimeric antigen receptor optimizes tonic signaling and CAR-T cell fitness. Cell Res. (2023) 33:341–54. doi: 10.1038/s41422-023-00789-0 36882513 PMC10156745

[64] Roddie C Sandhu KS Tholouli E Logan AC Shaughnessy P Barba P . Obecabtagene autoleucel in adults with B-cell acute lymphoblastic leukemia. N Engl J Med. (2024) 391:2219–30. doi: 10.1056/nejmoa2406526 39602653 PMC12818175

[65] Holzinger A Abken H . CAR T cells: a snapshot on the growing options to design a CAR. HemaSphere. (2019) 3. doi: 10.1097/HS9.0000000000000172 31723811 PMC6745938

[66] Carcopino C Erdogan E Henrich M Kobold S . Armoring chimeric antigen receptor (CAR) T cells as micropharmacies for cancer therapy. Immuno-Oncol Technol. (2024) 24. doi: 10.1016/j.iotech.2024.100739 39711794 PMC11659983

[67] Schelker RC Fioravanti J Mastrogiovanni F Baldwin JG Rana N Li P . LIM-domain-only 4 (LMO4) enhances CD8+ T-cell stemness and tumor rejection by boosting IL-21-STAT3 signaling. Signal Transduction Targeted Ther 2024 9:1. (2024) 9:199. doi: 10.1038/s41392-024-01915-z 39117617 PMC11310520

[68] Umland M Dragon AC Beermann LM Bonifacius A Kehler P Gellert J . Improved antitumor activity of interleukin-12-secreting chimeric antigen receptor T cells targeting CD176 across different carcinomas. Transfusion Med Hemotherapy. (2025) 53:1–15. doi: 10.1159/000549632 PMC12810983 41550609

[69] Nikolich-Žugich J . The twilight of immunity: emerging concepts in aging of the immune system. Nat Immunol. (2018) 19:10–9. doi: 10.1038/S41590-017-0006-X 29242543

[70] Zuo D Zhu Y Wang K Qin Y Su Y Lan S . A novel LAG3 neutralizing antibody improves cancer immunotherapy by dual inhibition of MHC-II and FGL1 ligand binding. Biomedicine Pharmacotherapy. (2024) 175. doi: 10.1016/j.biopha.2024.116782 38776682

[71] Annunziato F Manetti R Cosmi L Galli G Heusser CH Romagnani S . Opposite role for interleukin-4 and interferon-gamma on CD30 and lymphocyte activation gene-3 (LAG-3) expression by activated naive T cells. Eur J Immunol. (1997) 27:2239–44. doi: 10.1002/EJI.1830270918 9341765

[72] Sun H Sun C Xiao W . Expression regulation of co-inhibitory molecules on human natural killer cells in response to cytokine stimulations. Cytokine. (2014) 65:33–41. doi: 10.1016/j.cyto.2013.09.016 24139237

[73] Bhagwat AS Torres L Shestova O Shestov M Mellors PW Fisher HR . Cytokine-mediated CAR T therapy resistance in AML. Nat Med. (2024) 30:3697–708. doi: 10.1038/s41591-024-03271-5 39333315 PMC12118809

[74] Roederer M Nozzi JL Nason MC . SPICE: exploration and analysis of post-cytometric complex multivariate datasets. Cytometry Part A. (2011) 79 A:167–74. doi: 10.1002/CYTO.A.21015 21265010 PMC3072288

