# Supplementary material for: Impact of IL-21 on CAR T-cell manufacturing: phenotypic, metabolic, and functional outcomes
Source: Front Immunol. 2026 Aug 25;17:1819376. doi: 10.3389/fimmu.2026.1819376 (PMC13549998; doi:10.3389/fimmu.2026.1819376)
Supplement: Supplementary file 1 [file DataSheet1.pdf]

Supplementary Table 1: Patient characteristics and cytogenetic-molecular features of AML cases. AML diagnoses are reported according to the 2022 ICC and the 2022 WHO classifications. Pt: patient; PB: peripheral blood; ICC: International Consensus Classification; WHO: World Health Organization; BM: bone marrow.

Pt#	Sample	Disease Status	Diagnosis (ICC 2022//WHO 2022)	Molecular Biology and Cytogenetics	% Blasts in BM	PB lymphocyte count (cells/µL)	Age	Gender
1	PB (Cryopreserved)	Diagnosis	Acute myeloid leukemia with myelodysplasia-related gene mutations // Acute myeloid leukemia, myelodysplasia-related	Mutations: ASXL1, NF1, RUNX1, STAG2, TET2	32%	7790	87	Female
2	PB (Cryopreserved)	Diagnosis	Acute myeloid leukemia with mutated NPM1 // Acute myeloid leukemia with NPM1 mutation	Mutations: IDH2; NPM1; CEBPA	94%	14330	71	Female
3	PB (Cryopreserved)	Diagnosis	Acute myeloid leukemia with inv(3)(q21.3q26.2) or t(3;3)(q21.3;q26.2)/GATA2::MECOM // Acute myeloid leukemia with MECOM rearrangement	GATA2::MECOM fusion; inv(3)(q21q26)	52%	10600	41	Female
4	PB (Cryopreserved)	Diagnosis	Acute myeloid leukemia with inv(16)(p13.1q22) or t(16;16)(p13.1;q22)/CBFB::MYH11 // Acute myeloid leukemia with CBFB::MYH11 fusion	inv(16); CBFB::MYH11 rearrangement	86%	9600	72	Male
5	PB	Diagnosis	Acute myeloid leukemia with MECOM rearrangement // Acute myeloid leukemia with other MECOM rearrangements	Heterozygous TP53 deletion; MECOM::KLHL22 rearrangement; del7(q); subclonal deletions on chromosomes 5 and 17	34%	700	57	Male
6	PB	Diagnosis	Acute myeloid leukemia with mutated NPM1 // Acute myeloid leukemia with NPM1 mutation	Mutations: NPM1, CBL, TET2	88%	1100	73	Female

Supplementary Table 2: List of flow cytometry antibodies and staining reagents.

Antibody	Fluorochrome	Clone	Manufacturer	Reference	RRID
**Biotin-SP-AffiniPure Goat Anti-Mouse IgG, F(ab')$_2$**	NA	Polyclonal	Jackson Immuno Research	JAC115-065-072	RRID:AB_2338565
**Brilliant Stain Buffer**	NA	NA	BD Biosciences	566349	NA
**CD3**	PB	UCHT1	BD Biosciences	558117	RRID:AB_397038
**CD3**	FITC	SK7	BD Biosciences	345763	RRID:AB_2811220
**CD3**	APC	UCHT1	BD Biosciences	555335	RRID:AB_398591
**CD4**	PerCP-Cy5.5	SK3	BD Biosciences	566923	RRID:AB_2739678
**CD4**	APC-H7	RPA-T4	BD Biosciences	560158	RRID:AB_1645478
**CD45 (human)**	BV510	HI30	BD Biosciences	563204	RRID:AB_2738067
**CD45 (mouse)**	PE-Cy7	30-F11	BD Biosciences	552848	RRID:AB_394489
**CD8**	PE-Cy7	RPA-T8	BD Biosciences	557746	RRID:AB_396852
**CD8**	APC	RPA-T8	BD Biosciences	555369	RRID:AB_398595
**CD8**	APC-H7	SK1	BD Biosciences	560179	RRID:AB_1645481
**CD45RA**	PE-Cy7	HI100	BD Biosciences	568557	RRID:AB_3166166
**CD45RO**	BUV661	UCHL1	BD Biosciences	749887	RRID:AB_2874127
**CD62L**	BV510	DREG-56	BD Biosciences	563203	RRID:AB_2738066
**CD69**	PerCP-Cy5.5	FN50	BD Biosciences	560738	RRID:AB_1727510
**CD84**	PE	CD84.1.21	BioLegend	326008	RRID:AB_2229003
**CD95**	PE	DX2	BD Biosciences	556641	RRID:AB_396507
**Isotype Mouse IgG2a κ**	PE	MOPC-173	BioLegend	400214	RRID:AB_2800438
**CTLA-4 (CD152)**	PE	BNI3	BD Biosciences	557301	RRID:AB_396628
**LAG-3 (CD223)**	BV605	11C3C65	BioLegend	369324	RRID:AB_2721541
**PD-1 (CD279)**	PE-Cy7	EH12.1	BD Biosciences	561272	RRID:AB_10611585
**TIM-3 (CD366)**	BB515	7D3	BD Biosciences	565568	RRID:AB_2744368
**Streptavidin**	PE	NA	eBioscience	12-4317-87	NA
**Streptavidin**	BV421	NA	BD Biosciences	563259	NA

NA: not applicable

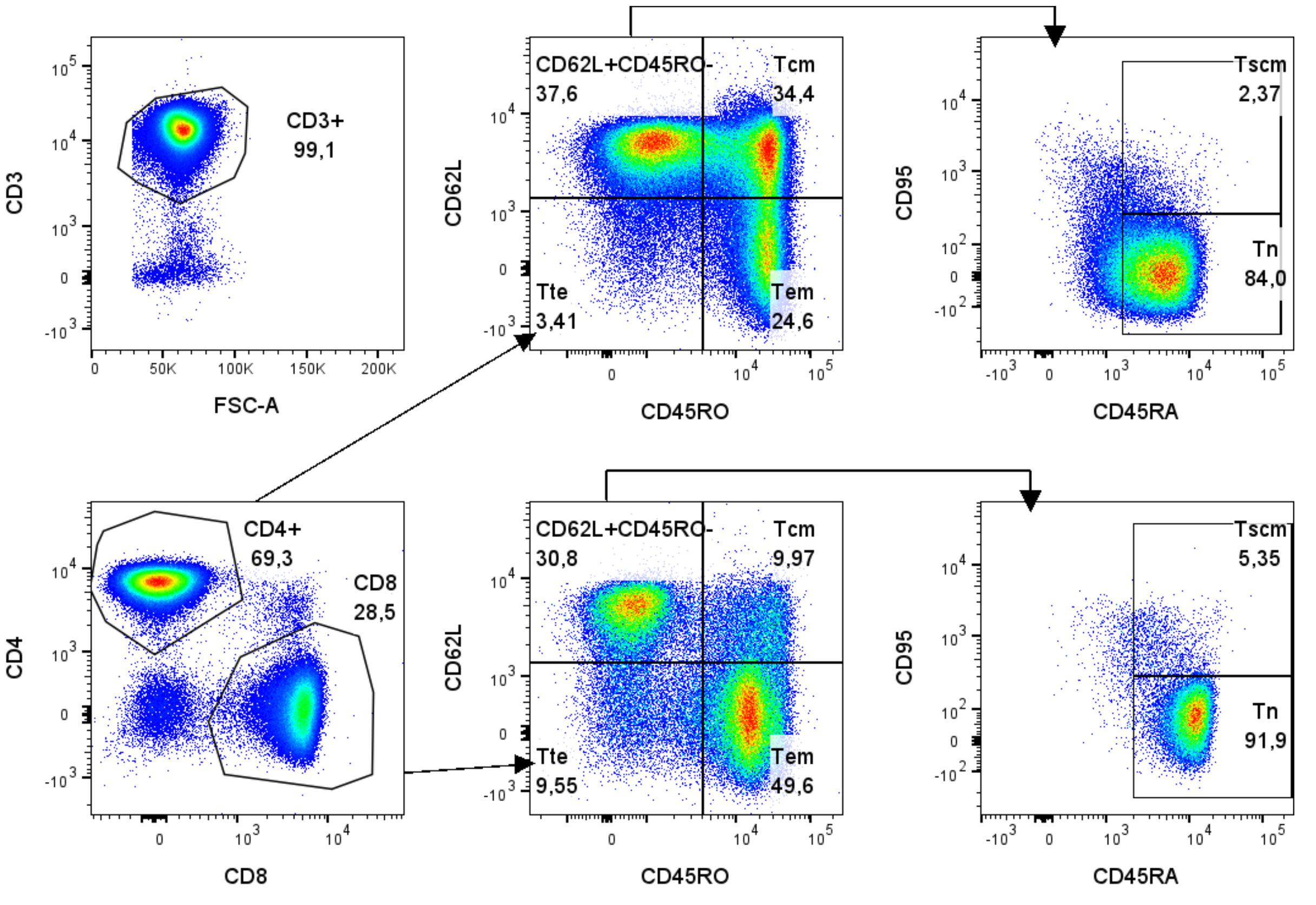

Supplementary Figure 1: Gating strategy for the phenotypic characterization of T-cell subsets in CART84. First, $CD3^+$ T lymphocytes were identified, which were then subdivided into $CD4^+$ and $CD8^+$ T-cell populations. Within each subset, naïve T cells (T_N; $CD62L^+CD45RO^-CD45RA^+CD95^-$), stem cell memory T cells (T_{SCM}; $CD62L^+CD45RO^-CD45RA^+ CD95^+$), central memory T cells (T_{CM}; $CD62L^+CD45RO^+$), effector memory T cells (T_{EM}; $CD62L^-CD45RO^+$), and terminal effector T cells (T_{TE}; $CD62L^-CD45RO^-$) were distinguished.

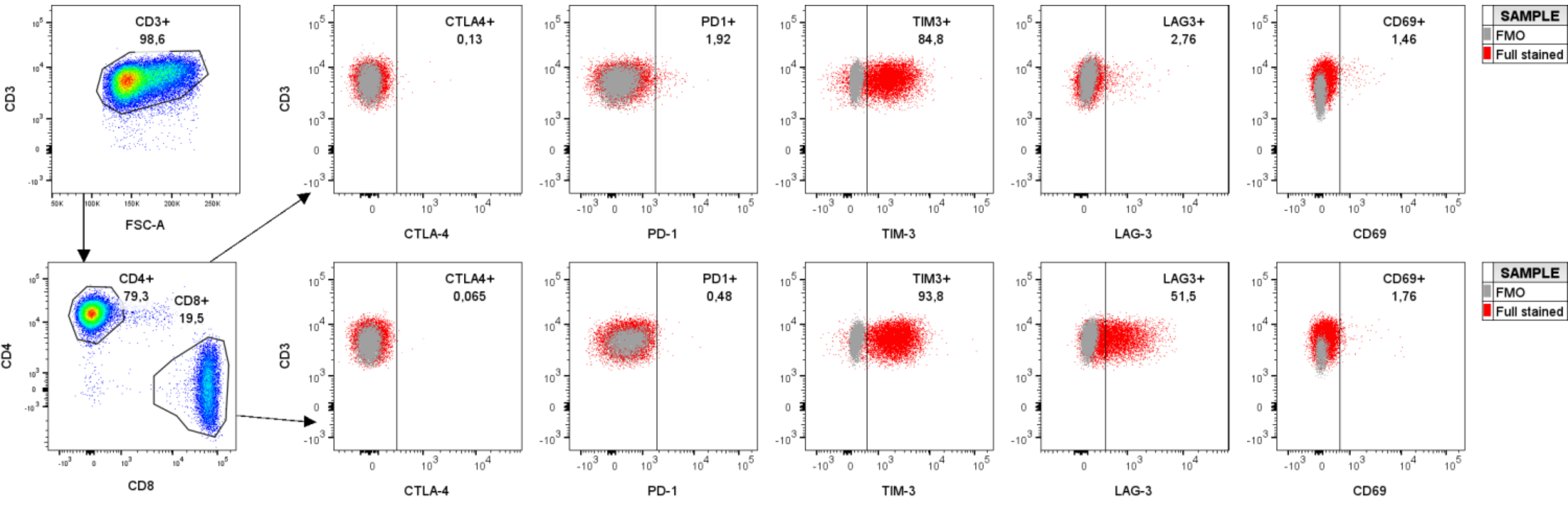

Supplementary Figure 2: Gating strategy for the analysis of exhaustion- and activation-associated marker expression. $CD3^+$ T lymphocytes were first identified and subsequently subdivided into $CD4^+$ and $CD8^+$ T cell populations. Within each subset, the frequency of cells positive for CTLA-4, PD-1, TIM-3, LAG-3, and CD69 was determined. Positivity thresholds were defined using fluorescence minus one (FMO) controls and applied consistently across experimental samples (IL-2, IL-7+15, or IL-7+15+21) within each donor.

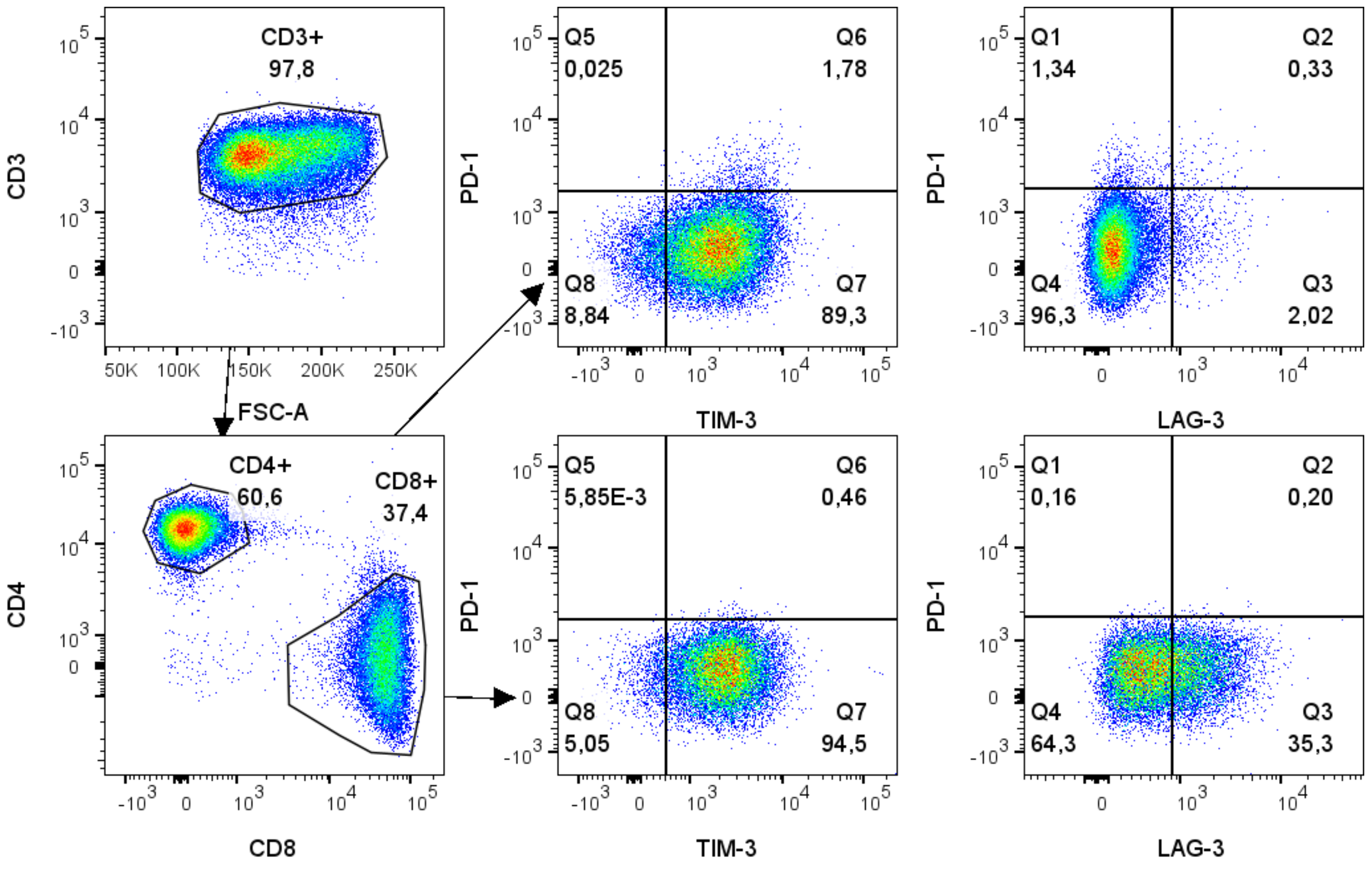

Supplementary Figure 3: Gating strategy for the identification of double-positive PD-1^{+}TIM-3^{+} and PD-1^{+}LAG-3^{+} populations. CD3^{+} T lymphocytes were first identified and subsequently subdivided into CD4^{+} and CD8^{+} T-cell populations. Within each subset, the percentage of PD-1^{+}TIM-3^{+} (Q6) and PD-1^{+}LAG-3^{+} (Q2) populations were quantified.

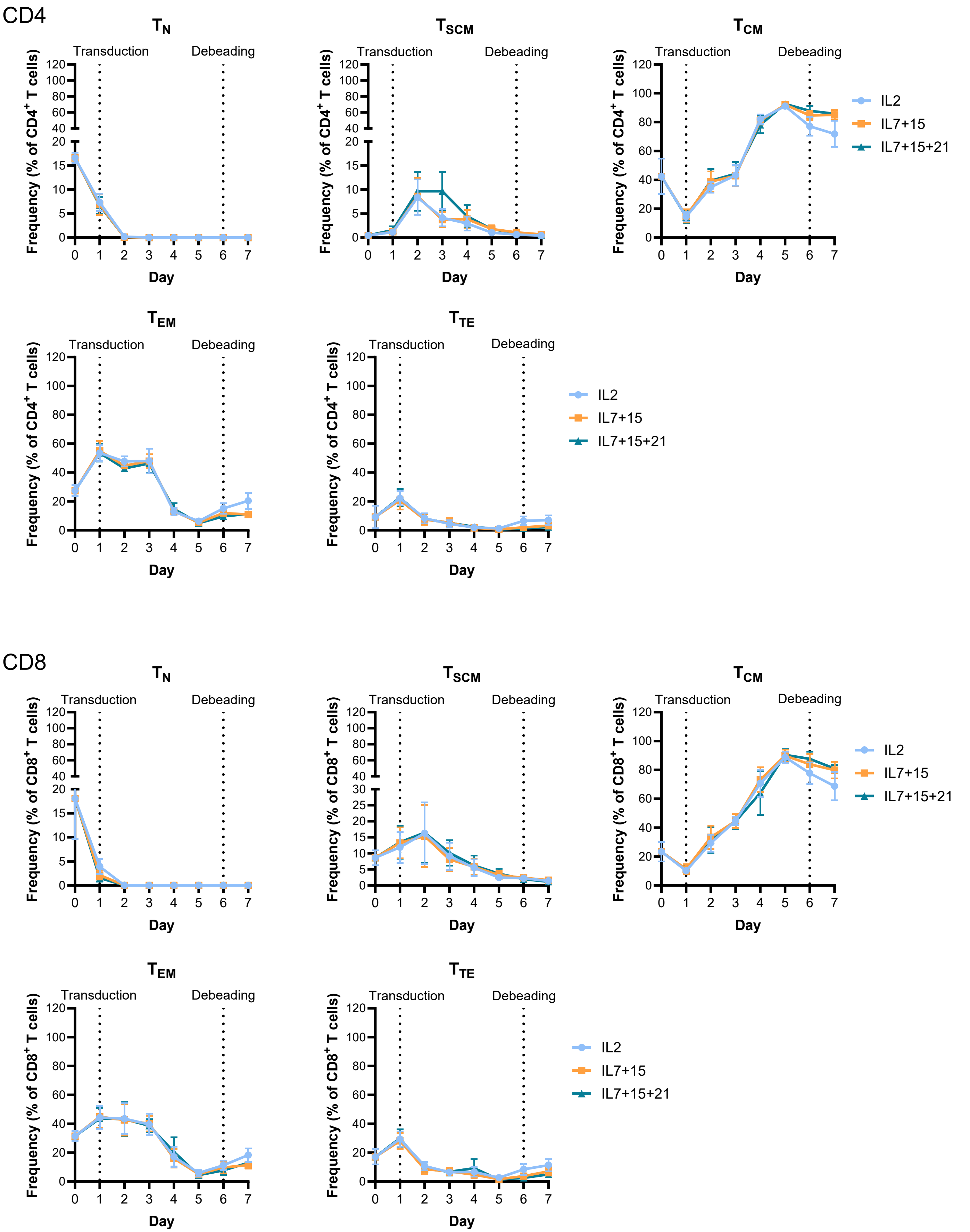

Supplementary Figure 4: Phenotypic characterization of T-cell subset kinetics in CART84 expanded under different cytokine conditions. Naïve T cells (T_N), stem cell memory T cells (T_{SCM}), central memory T cells (T_{CM}), effector memory T cells (T_{EM}), and terminal effector T cells (T_{TE}) were identified within CD4⁺ (top) and CD8⁺ (bottom) T cells using the gating strategy described in Supplementary Figure 1. Data represent 3 independent donors (n = 3), mean ± S.E.M.

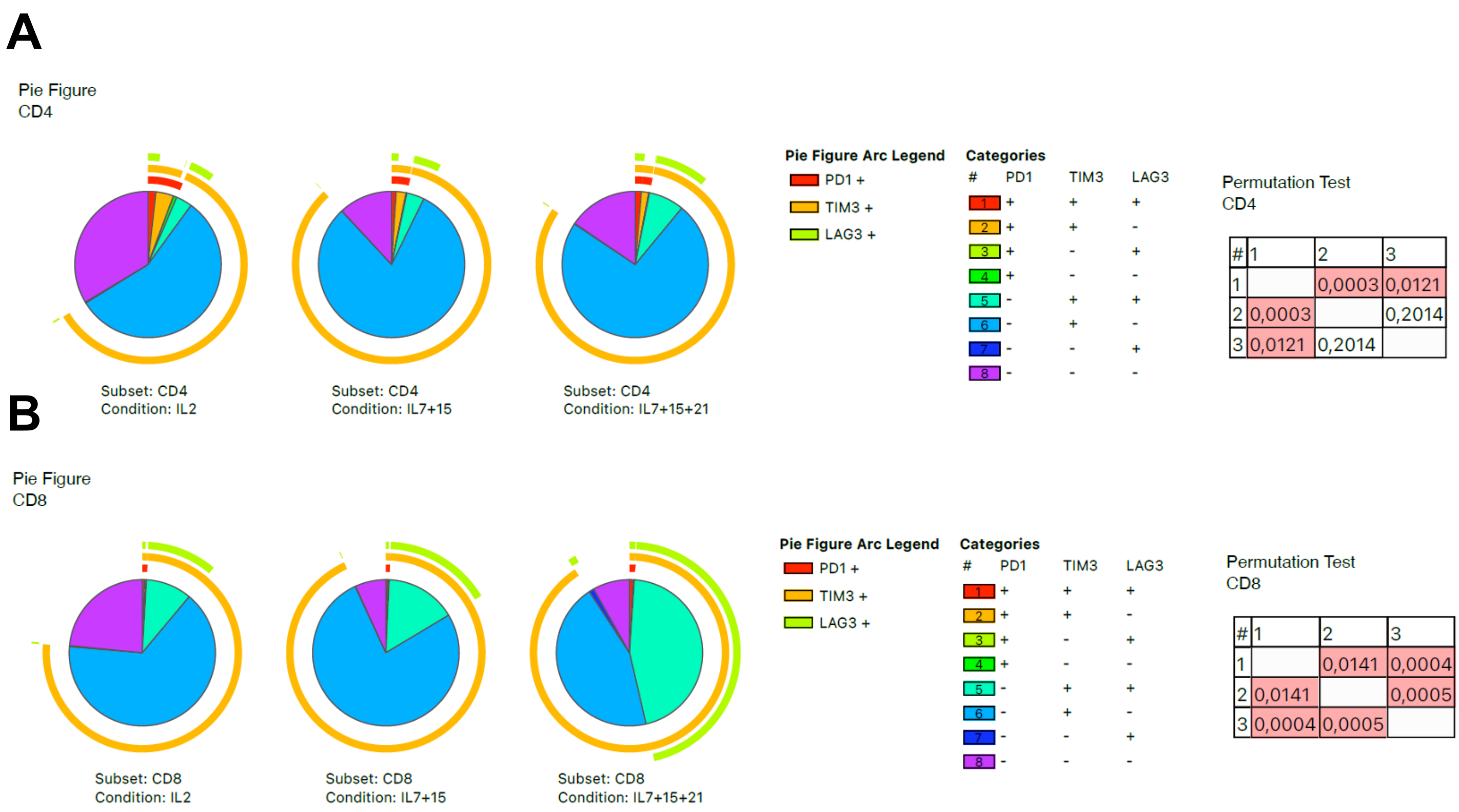

Supplementary Figure 5: Simplified Presentation of Incredibly Complex Evaluations (SPICE) analysis of PD-1, LAG-3, and TIM-3 co-expression patterns in CD4$^+$ **(A)** and CD8$^+$ **(B)** CART84 cells expanded under different cytokine conditions. FlowJo gating was performed as described in Supplementary Figure 2, and combinatorial PD-1, LAG-3, and TIM-3 expression gates were exported to SPICE for visualization and analysis. Pie charts depict the relative distribution of marker co-expression patterns across conditions. Permutation test results indicating overall differences in population distributions between conditions are shown (1 = IL-2; 2 = IL-7+15; 3 = IL-7+15+21). Data represent the mean of n = 9 independent donors (same donors across all panels). Statistical significance, highlighted in red, was set at $p < 0.05$.

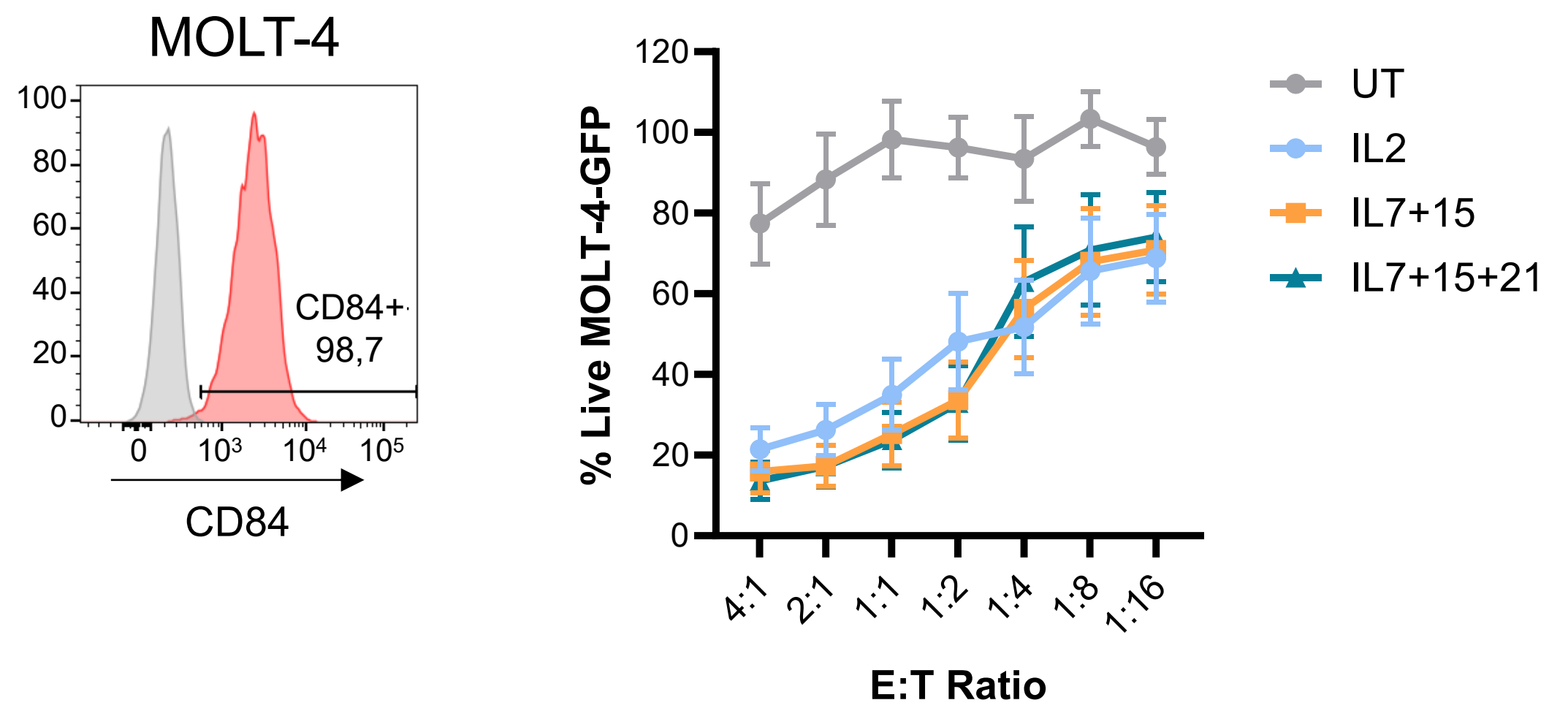

Supplementary Figure 6: Cytotoxicity assays of CART84 expanded under different cytokine conditions against MOLT-4. Untransduced (UT) T cells and CART84 generated under the indicated cytokine conditions were co-cultured with the T-ALL cell line MOLT-4 for 24 h at the indicated effector-to-target (E:T) ratios. Representative flow cytometry histograms showing CD84 expression (red) relative to isotype control (grey) in MOLT-4 cells are shown. Cytotoxicity was analyzed using a two-way ANOVA followed by Tukey's post-test for multiple comparisons. Only CART84 cells expanded under different cytokine conditions were included in the statistical analysis (UT excluded). Data represent 4-7 independent donors (n = 4–7), mean ± S.E.M. Comparisons not reaching significance are not shown.

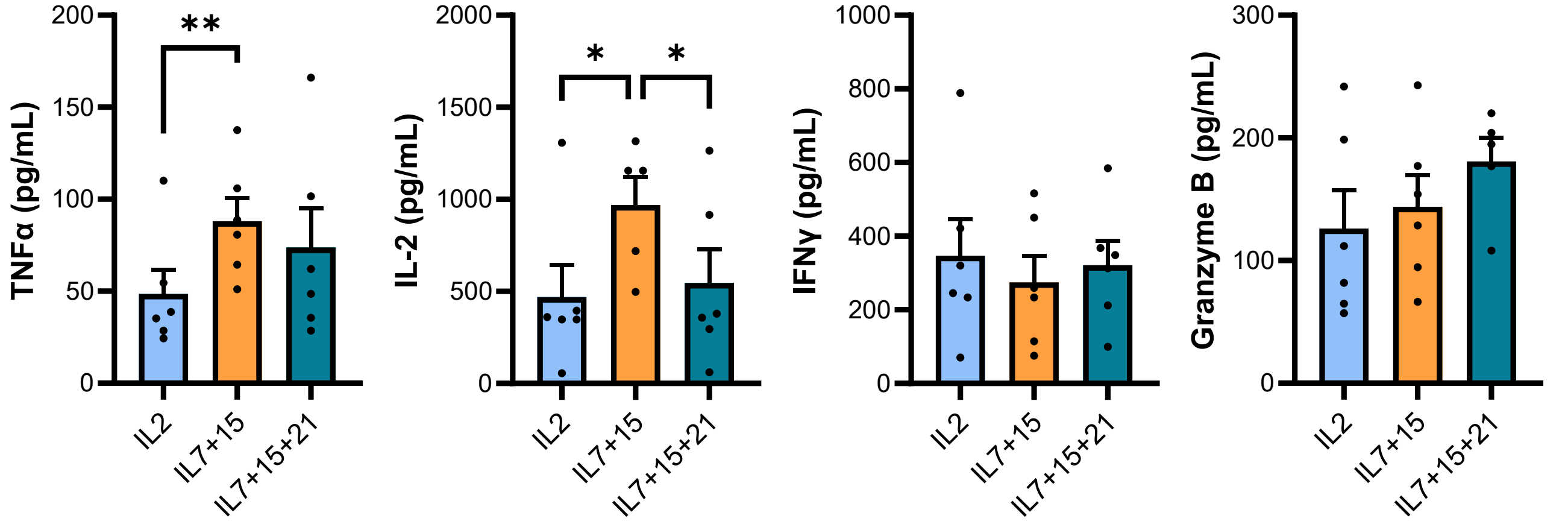

Supplementary Figure 7: Cytokine production by CART84 expanded under different cytokine conditions during co-culture with MOLM-13. CART84 generated under the indicated cytokine conditions were co-cultured with MOLM-13 cells for 24 h as in Figure 3. Cell-free supernatants of the 1:2 effector-to-target ratio were harvested and TNFα, IL-2, IFNγ, and granzyme B levels were quantified by ELISA. Outliers were identified using the ROUT method (Q = 1%) and excluded from subsequent analyses. One granzyme B value in the IL-7+15+21 condition was excluded as an outlier. In addition, one IL-2 measurement from the IL-7+15 condition could not be quantified because it exceeded the assay detection range. Statistical analysis was performed using one-way ANOVA, or mixed-effects models when values were missing, with donor-matched cytokine conditions (IL-2, IL-7+15 and IL-7+15+21), followed by Tukey's post-test for multiple comparisons. Data represent six independent donors (n = 6; same donors across all panels), mean ± S.E.M. *: $p < 0.05$; **: $p < 0.01$. Comparisons not reaching significance are not shown.

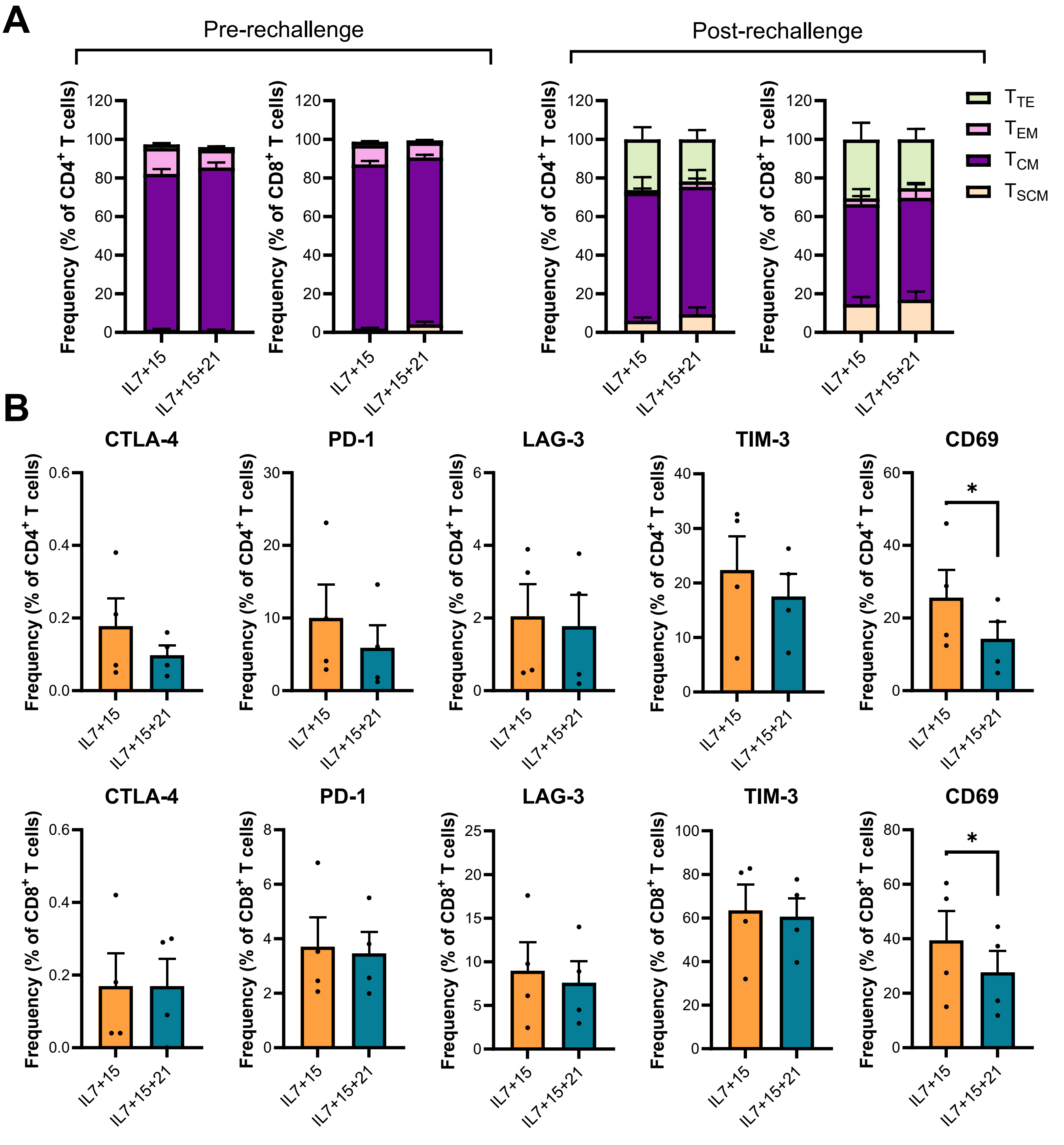

Supplementary Figure 8: Characterization of CART84 expanded with IL-7+15 or IL-7+15+21 before (pre-rechallenge, day 0) and after (post-rechallenge, day 9) repeated antigen exposure experiment in Figure 5. **(A)** Phenotypic characterization of T-cell memory subsets within CD4⁺ and CD8⁺ T cells before (pre-rechallenge) and after (post-rechallenge) repeated antigen exposure, using the gating strategy shown in Supplementary Figure 1. Statistical analysis was performed using two-way ANOVA with donor-matched cytokine conditions (IL-7+15 and IL-7+15+21) and memory populations, followed by Sídák's post-test for multiple comparisons. **(B)** Percentage of cells positive for the indicated exhaustion and activation markers within CD4⁺ (top row) and CD8⁺ (bottom row) T cells at the end of the experiment, using the gating strategy shown in Supplementary Figure 2. Statistical analysis was performed using a paired t test. Data represent 4 independent donors (n = 4; same donors across all panels), mean ± S.E.M. *: $p < 0.05$, **: $p < 0.01$. Comparisons not reaching significance are not shown.

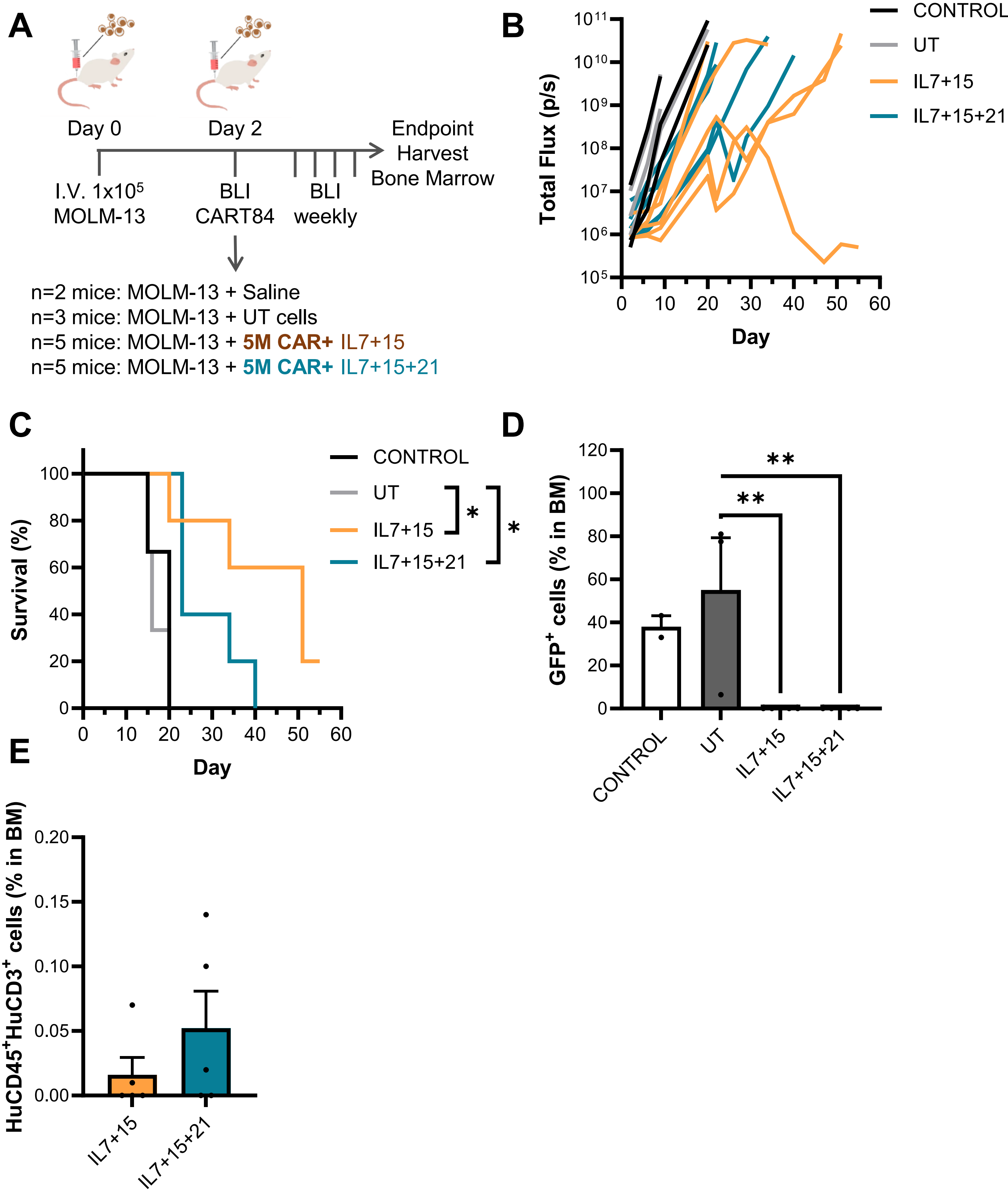
A
Day 0
Day 2
Endpoint
Harvest
Bone Marrow
I.V. 1x10⁵
MOLM-13
BLI
CART84
BLI
weekly
n=2 mice: MOLM-13 + Saline
n=3 mice: MOLM-13 + UT cells
n=5 mice: MOLM-13 + 5M CAR+ IL7+15
n=5 mice: MOLM-13 + 5M CAR+ IL7+15+21
B
Total Flux (p/s)
Day
CONTROL
UT
IL7+15
IL7+15+21
C
Survival (%)
Day
CONTROL
UT
IL7+15
IL7+15+21
*
*
D
GFP⁺ cells (% in BM)
**
**
CONTROL
UT
IL7+15
IL7+15+21
E
HuCD45⁺HuCD3⁺ cells (% in BM)
IL7+15
IL7+15+21

Supplementary Figure 9: Effect of IL-21 addition on the in vivo efficacy of CART84. **(A)** Experimental design: on day 0, 1×10^5 MOLM-13-GFP-ffLuc cells were administered intravenously (i.v.) to immunodeficient NSG male mice. On day 2, mice were allocated to experimental groups based on tumor burden to ensure similar baseline tumor loads. Subsequently, $5\text{x}10^6$ cryopreserved CAR^+ CART84 cells expanded with IL-7+15 or IL-7+15+21, untransduced (UT) cells, or saline were administered i.v. Leukemia burden was monitored weekly by bioluminescence imaging (BLI). Mice were euthanized upon reaching humane endpoint criteria or at experimental endpoint, and bone marrow was collected at sacrifice for flow cytometry analysis. The number of mice per group is indicated in the experimental design. All cryopreserved UT and CART84 cells were generated from a single healthy donor. **(B)** Leukemia burden was quantified by bioluminescence imaging (BLI; total flux, photons/s). Statistical analysis was performed using mixed-effects models with matching by time point, followed by Tukey's post-test for multiple comparisons. The control group (n = 2 mice) was excluded from the statistical analysis; comparisons were limited to UT and CART84 cells. Comparisons not reaching significance are not shown. **(C)** Kaplan–Meier survival curves. Statistical analysis was performed using a log-rank (Mantel–Cox) test with p-value correction for three comparisons (UT vs. IL-7+15, UT vs IL-7+15+21, and IL-7+15 vs IL-7+15+21). *: $p < 0.017$. Comparisons not reaching significance are not shown. **(D)** Frequency (%) of GFP^+ tumor cells in bone marrow (BM) at endpoint (following humane endpoint criteria or experimental endpoint). Statistical analysis was performed using a one-way ANOVA followed by Tukey's post-test for multiple comparisons. The control group (n = 2 mice) was excluded from the statistical analysis; comparisons were limited to UT and CART84 cells. Mean ± S.E.M. **: $p < 0.01$. Comparisons not reaching significance are not shown. **(E)** Frequency (%) of CART84 cells in the bone marrow (BM) at endpoint (following humane endpoint criteria or experimental endpoint). CART84 were identified as human $CD45^+$ and human $CD3^+$ cells ($HuCD45^+HuCD3^+$). Statistical analysis was performed using an unpaired t test. Mean ± S.E.M. Comparisons not reaching significance are not shown.

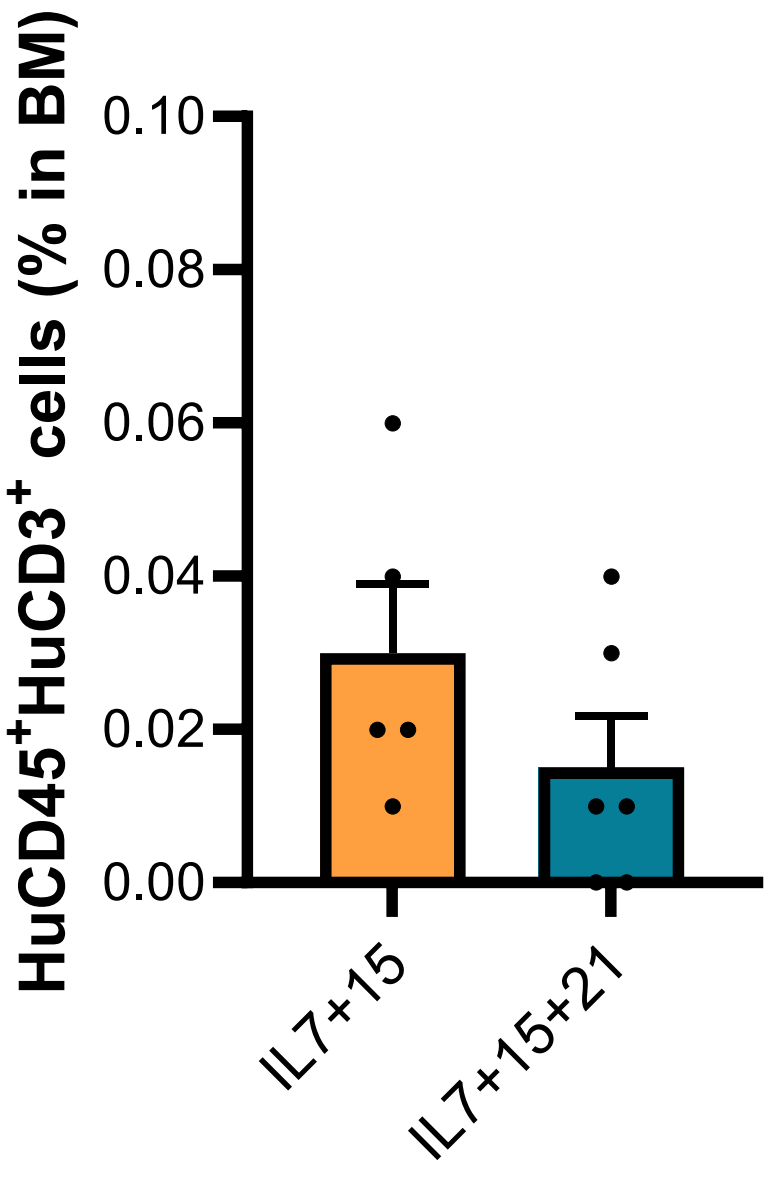

Supplementary Figure 10: Frequency (%) of CART84 cells in the bone marrow (BM) at endpoint (following humane endpoint criteria or experimental endpoint) from mice shown in Figure 7. CART84 were identified as human $CD45^+$ and human $CD3^+$ cells ($HuCD45^+HuCD3^+$). Statistical analysis was performed using an unpaired t test. Data represent n = 5 mice for IL-7+15, n = 6 mice for IL-7+15+21, mean ± S.E.M. Comparisons not reaching significance are not shown.

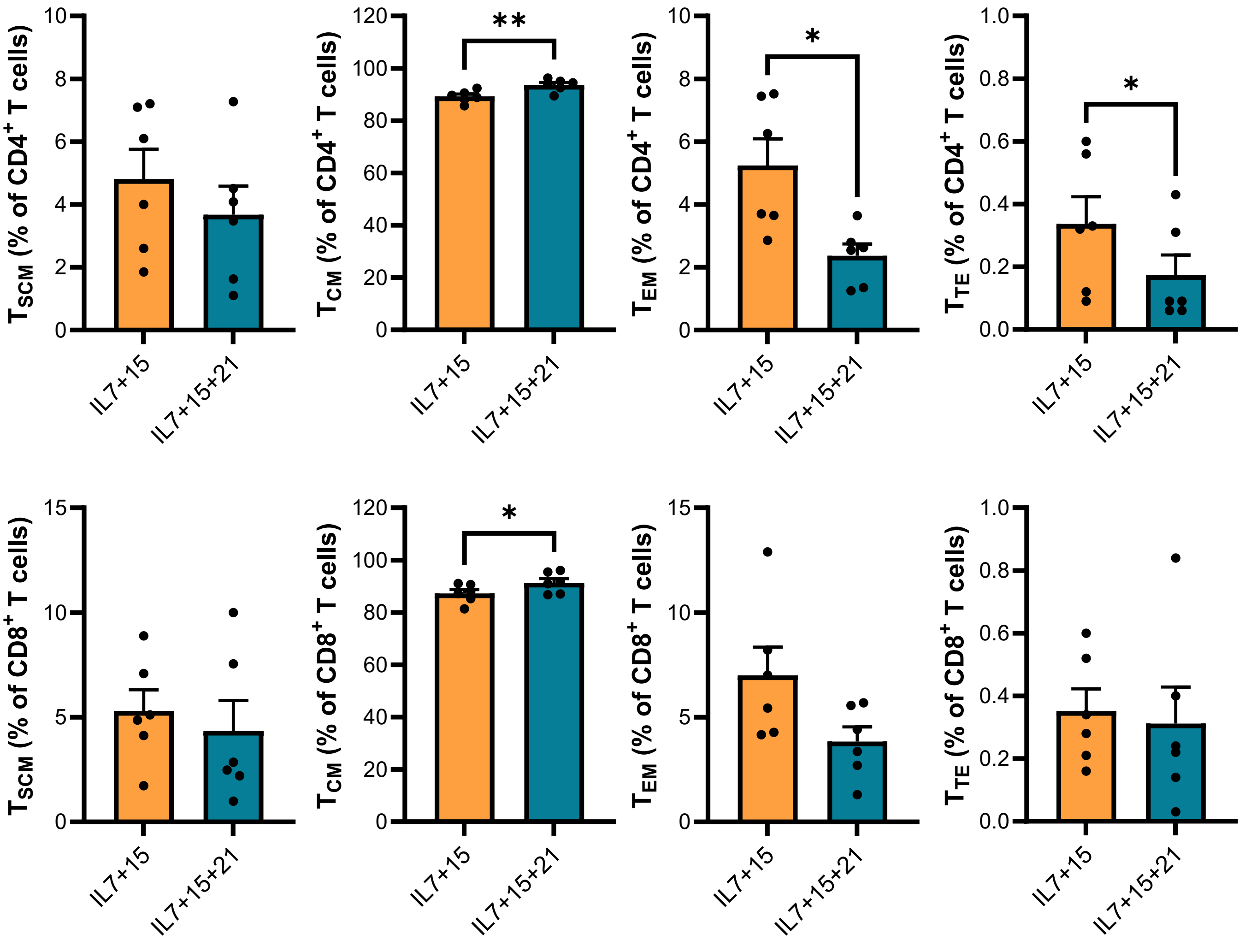

Supplementary Figure 11: Phenotypic characterization of T-cell memory subsets in G-Rex-expanded CART84 with or without IL-21. Naïve T cells (T_N), stem cell memory T cells (T_{SCM}), central memory T cells (T_{CM}), effector memory T cells (T_{EM}), and terminal effector T cells (T_{TE}) were identified within $CD4^+$ (top row) and $CD8^+$ (bottom row) T-cells using the gating strategy described in Supplementary Figure 1. T_N cells are not shown due to their negligible frequency at this time point. Statistical analysis was performed using a paired t test. The ROUT method (Q = 1%) was used to identify outliers; no values were excluded from the analysis. Data represent 6 independent donors (n = 6; same donors across all panels), mean ± S.E.M. *: $p < 0.05$, **: $p < 0.01$. Comparisons not reaching significance are not shown.

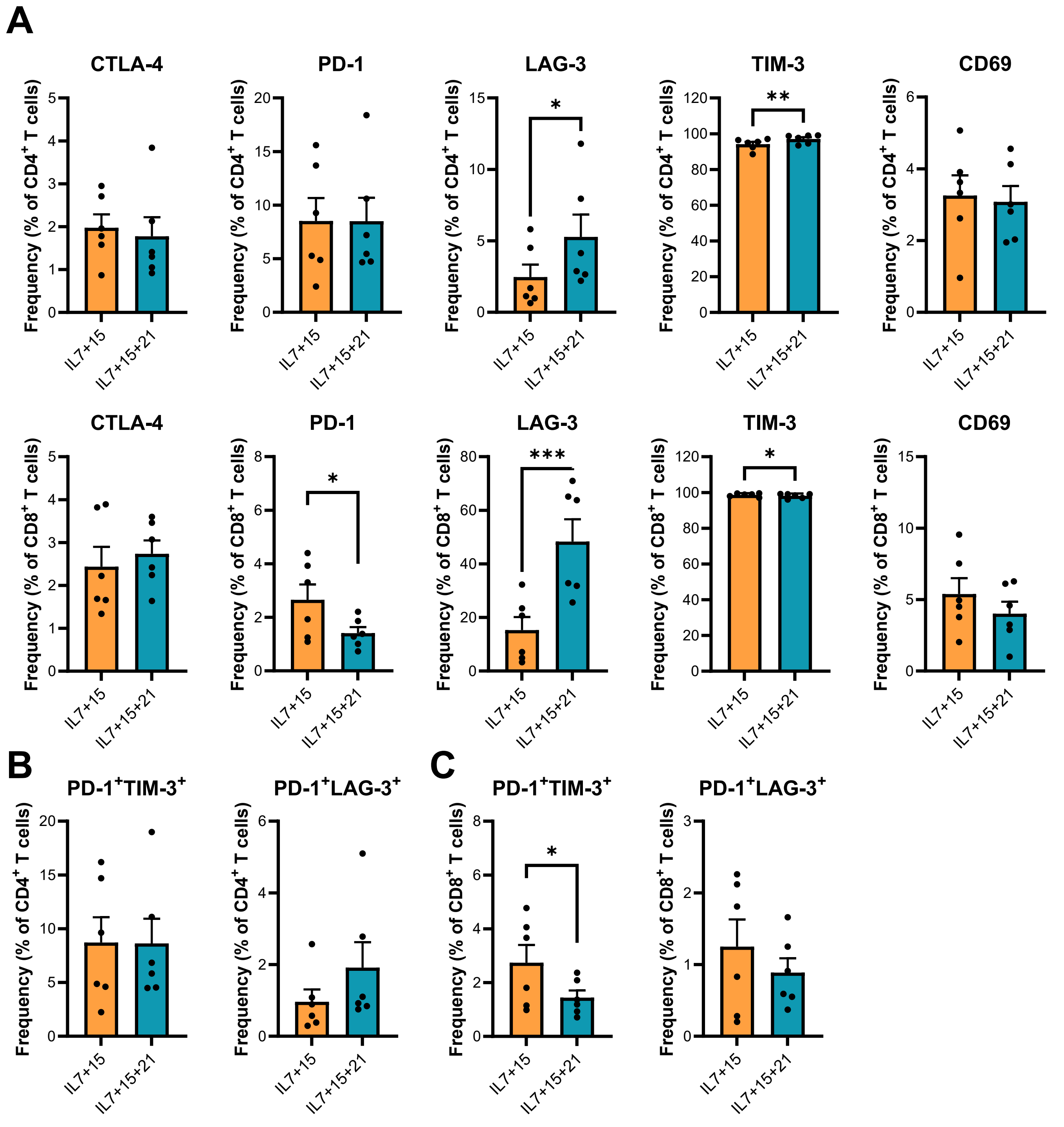

Supplementary Figure 12: Exhaustion- and activation-associated marker expression in G-Rex-expanded CART84 cells with or without IL-21. **(A)** Percentage of cells positive for the indicated exhaustion and activation markers within $CD4^+$ (top row) and $CD8^+$ (bottom row) T-cell subsets. Gates for marker positivity were defined using fluorescence minus one (FMO) controls, as described in Supplementary Figure 2. **(B-C)** Percentage of $PD\text{-}1^+TIM\text{-}3^+$ and $PD\text{-}1^+LAG\text{-}3^+$ double-positive cells within $CD4^+$ **(B)** and $CD8^+$ **(C)** T-cell subsets, using the gating strategy shown in Supplementary Figure 3. Statistical analysis was performed using a paired t test. Data represent 6 independent donors (n = 6; same donors across all panels), mean ± S.E.M. *: $p < 0.05$, **: $p < 0.01$, ***: $p < 0.001$. Comparisons not reaching significance are not shown.

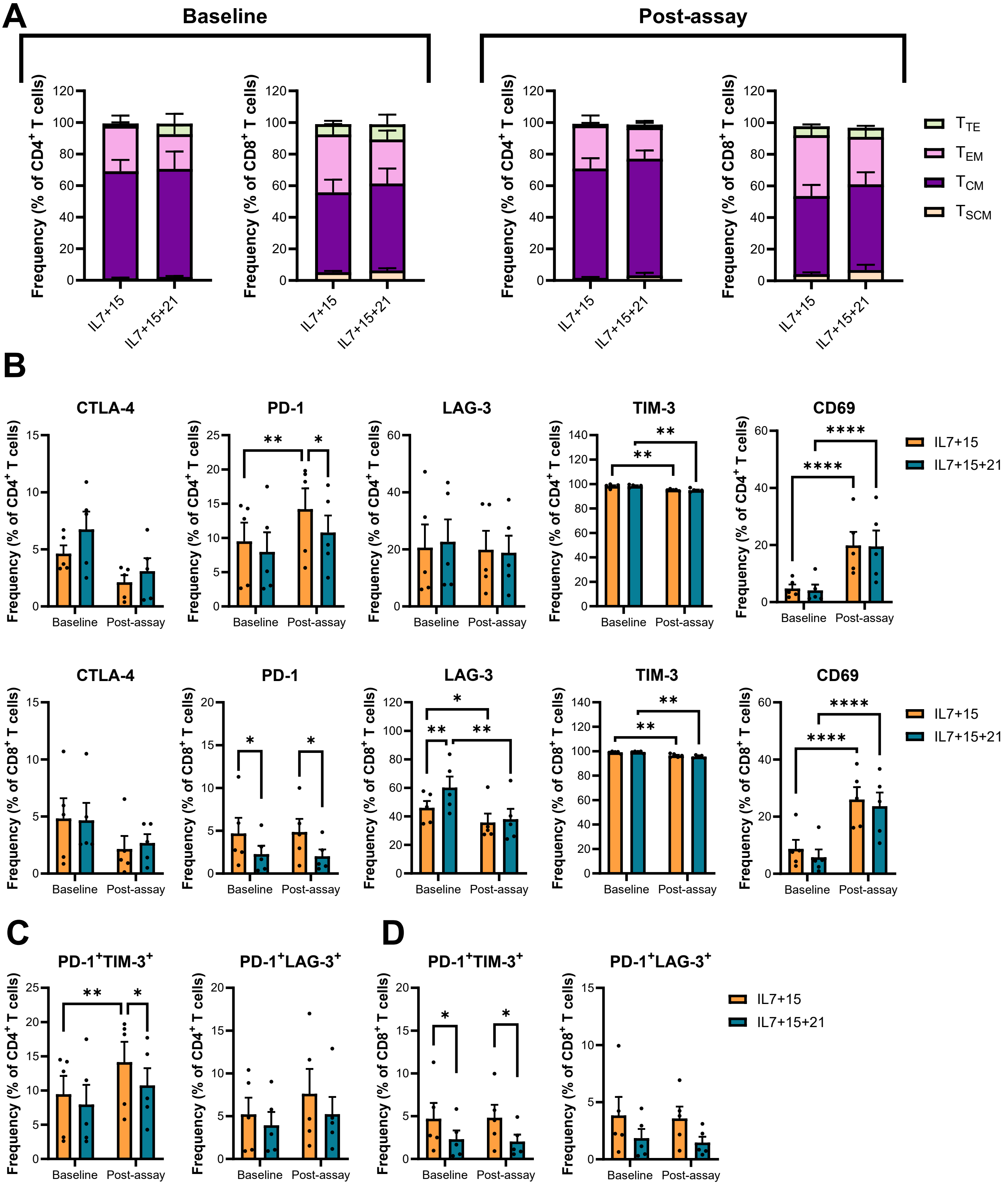
A
Baseline
Post-assay
Frequency (% of CD4+ T cells)
Frequency (% of CD8+ T cells)
IL7+15
IL7+15+21
TTE
TEM
TCM
TSCM
B
CTLA-4
PD-1
LAG-3
TIM-3
CD69
Baseline
Post-assay
IL7+15
IL7+15+21
C
PD-1+TIM-3+
PD-1+LAG-3+
D
PD-1+TIM-3+
PD-1+LAG-3+

Supplementary Figure 13: Phenotypic characterization of CART84 cells generated from AML patient-derived T cells, expanded with or without IL-21 supplementation, assessed at the end of CART84 expansion (baseline) and after the cytotoxicity assay against MOLM-13 cells shown in Figure 9D (post-assay). **(A)** Distribution of T-cell memory subsets within $CD4^+$ and $CD8^+$ CART84 cells at baseline and post-assay. Memory subsets were defined as described in Supplementary Figure 1. Statistical analysis was performed using two-way ANOVA with donor-matched cytokine conditions (IL-7+15 and IL-7+15+21) and memory subsets, followed by Šídák's post-test for multiple comparisons. Data represent 6 independent patients (n = 6), mean ± S.E.M. **(B)** Frequency of cells positive for the indicated activation and exhaustion markers within $CD4^+$ (top row) and $CD8^+$ (bottom row) T-cell subsets at baseline and post-assay. Gates for marker positivity were defined using fluorescence minus one (FMO) controls, as described in Supplementary Figure 2. Statistical analysis was performed using two-way ANOVA with donor-matched cytokine conditions (IL-7+15 and IL-7+15+21) and time point (baseline and post-assay), followed by Šídák's post-test for multiple comparisons. Data represent 5 independent patients (n = 5), mean ± S.E.M. **(C-D)** Percentage of PD-1^+TIM-3^+ and PD-1^+LAG-3^+ double-positive cells within $CD4^+$ **(C)** and $CD8^+$ **(D)** T-cell subsets at baseline and post-assay, using the gating strategy shown in Supplementary Figure 3. Statistical analysis was performed using two-way ANOVA with donor-matched cytokine conditions (IL-7+15 and IL-7+15+21) and time point (baseline and post-assay), followed by Sídák's post-test for multiple comparisons. Only comparisons between cytokine conditions (IL-7+15 vs IL-7+15+21) or between baseline and post-assay within each condition are shown. Data represent 5 independent patients (n = 5), mean ± S.E.M. *: $p < 0.05$, **: $p < 0.01$, ***: $p < 0.001$, ****: $p < 0.0001$. Comparisons not reaching significance are not shown.
